# Glycerol Kinase Drives Hepatic de novo Lipogenesis and Triglyceride Synthesis in Nonalcoholic Fatty Liver by Activating *SREBP‐1c* Transcription, Upregulating DGAT1/2 Expression, and Promoting Glycerol Metabolism

**DOI:** 10.1002/advs.202401311

**Published:** 2024-10-17

**Authors:** Shuyu Ouyang, Shu Zhuo, Mengmei Yang, Tengfei Zhu, Shuting Yu, Yu Li, Hao Ying, Yingying Le

**Affiliations:** ^1^ CAS Key Laboratory of Nutrition Metabolism and Food Safety Shanghai Institute of Nutrition and Health University of Chinese Academy of Sciences Chinese Academy of Sciences Shanghai 200031 China; ^2^ School of Public Health Shanghai Jiao Tong University School of Medicine Shanghai 200025 China

**Keywords:** de novo lipogenesis, glycerol kinase, nonalcoholic fatty liver disease, SREBP‐1c, triglyceride

## Abstract

Glycerol kinase (GK) participates in triglyceride (TG) synthesis by catalyzing glycerol metabolism. Whether GK contributes to nonalcoholic fatty liver (NAFL) is unclear. The expression of hepatic Gk is found to be increased in diet‐induced and genetic mouse models of NAFL and is positively associated with hepatic SREBP‐1c expression and TG levels. Cholesterol and fatty acids stimulate GK expression in hepatocytes. In HFD‐induced NAFL mice, knockdown of hepatic Gk decreases expression of SREBP‐1c and its target lipogenic genes as well as DGAT1/2, increases serum glycerol levels, decreases serum TG levels, and attenuates hepatic TG accumulation. Overexpression of GK in hepatocytes in mice or in culture produces opposite results. Mechanistic studies reveal that GK stimulates SREBP‐1c transcription directly by binding to its gene promoter and indirectly by binding to SREBP‐1c protein, thereby increasing lipogenic gene expression and de novo lipogenesis. Studies with truncated GK and mutant GKs indicate that GK induces SREBP‐1c transcription independently of its enzyme activity. GK contributes to lipid homeostasis under physiological conditions by catalyzing glycerol metabolism rather than by regulating SREBP‐1c transcription. Collectively, these results demonstrate that increased hepatic GK promotes de novo lipogenesis and TG synthesis in NAFL by stimulating SREBP‐1c transcription and DGAT1/2 expression and catalyzing glycerol metabolism.

## Graphical abstract

This work found that glycerol kinase (GK) is upregulated in diet‐induced and genetic mouse models of nonalcoholic fatty liver (NAFL). Fatty acids and cholesterol stimulate GK expression in hepatocytes. Increased hepatic GK promotes de novo lipogenesis and TG synthesis during NAFL development by stimulating SREBP‐1c transcription and DGAT1/2 expression and catalyzing glycerol metabolism.

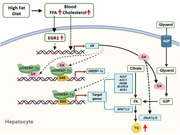

## 1 Introduction

Nonalcoholic fatty liver disease (NAFLD) is currently the leading cause of chronic liver disease worldwide. It comprises a spectrum of diseases that includes non‐alcoholic fatty liver (NAFL) and non‐alcoholic steatohepatitis (NASH).^[^ ^1^ ^]^ NAFL increases the risk of metabolic disorders, such as type 2 diabetes and hyperlipidemia, and can progress to NASH, liver cirrhosis, or hepatocellular carcinoma.^[^ ^1^ ^]^ Excessive accumulation of lipids (mainly triglycerides (TG)) in hepatocytes is the hallmark of NAFL. Studies using stable isotope tracing strategies demonstrated that the TG accumulated in the liver of NAFL patients was largely derived from de novo lipogenesis.^[^ ^2^, ^3^, ^4^ ^]^

Sterol regulatory element binding protein 1c (SREBP‐1c) is a major transcription factor that regulates the expression of enzymes involved in de novo lipogenesis and TG synthesis in hepatocytes, including ATP citrate lyase (ACLY), acetyl‐CoA carboxylase (ACC), fatty acid synthase (FASN), long‐chain elongase (ELOVL6), and stearoyl‐CoA desaturase 1 (SCD1) for fatty acid synthesis and desaturation; as well as glycerol‐3‐phosphate acyltransferase (GPAT) for TG synthesis.^[^ ^5^, ^6^ ^]^ SREBP‐1c protein is synthesized as inactive precursors residing at the endoplasmic reticulum membrane. After proteolytic cleavage, the N‐terminal domain of the precursor translocates to the nucleus to activate its own gene and other target genes by binding to the sterol regulatory element (SRE) in their promoters. The expressions of SREBP‐1c and its target lipogenic genes are increased in the livers of human NAFLD patients^[^ ^7^, ^8^ ^]^ and animal models of NAFL.^[^ ^9^, ^10^ ^]^ Knockdown or inhibition of SREBP‐1c or its target lipogenic enzymes, such as ACC, SCD1, ELOVL6, and GPAT, reduced hepatic lipid deposition in NAFLD animals and patients,^[^ ^11^, ^12^, ^13^ ^]^ indicating the critical contribution of SREBP‐1c to hepatic steatosis. The regulation of gene transcription, precursor protein processing, and protein posttranslational modification of SREBP‐1c under physiological conditions has been extensively studied.^[^ ^13^, ^14^ ^]^ However, the transcriptional regulation of SREBP‐1c in NAFL is not fully understood.

Glycerol kinase (GK) belongs to the FGGY kinase family, it catalyzes the phosphorylation of glycerol to glycerol‐3‐phosphate (G3P) by ATP. G3P is a substrate for TG synthesis and is involved in gluconeogenesis. Our previous study showed that GK regulates hepatic gluconeogenesis not only by converting glycerol to G3P, but also by upregulating the expression of phosphoenolpyruvate carboxykinase and glucose‐6‐phosphatase through the AKT‐FOXO1 pathway. GK plays an important role in glucose homeostasis under physiological conditions and contributes to hyperglycemia in diabetic mice.^[^ ^15^ ^]^ We found that hepatic GK was upregulated in HFD‐induced obese mice with hyperglycemia and hyperlipidemia.^[^ ^15^ ^]^ However, it's not clear whether GK contributes to lipid homeostasis under physiological and to the development of NAFL.

In this study, we showed that GK is upregulated in the livers of diet‐induced and genetic mouse models of NAFL and plays a crucial role in the development of hepatic steatosis. We identified cholesterol and fatty acid as regulators of GK expression in hepatocytes. Through adenovirus‐mediated gain‐ and loss‐of‐function studies, we demonstrated that GK not only participates in hepatic TG synthesis by catalyzing glycerol metabolism but also promotes hepatic de novo lipogenesis and TG synthesis by upregulating SREBP‐1c transcription. We further explored the molecular mechanisms underlying the regulation of SREBP‐1c transcription by GK. Additionally, we investigated the contribution of hepatic GK to lipid homeostasis under physiological conditions.

## 2 Results

### 2.1 Knockdown of Hepatic *Gk* Alleviates HFD‐Induced Hepatic Steatosis and Reduces the Expression of *Srebp‐1c* and its Target Lipogenic Genes in Mice

**Figure 1:**
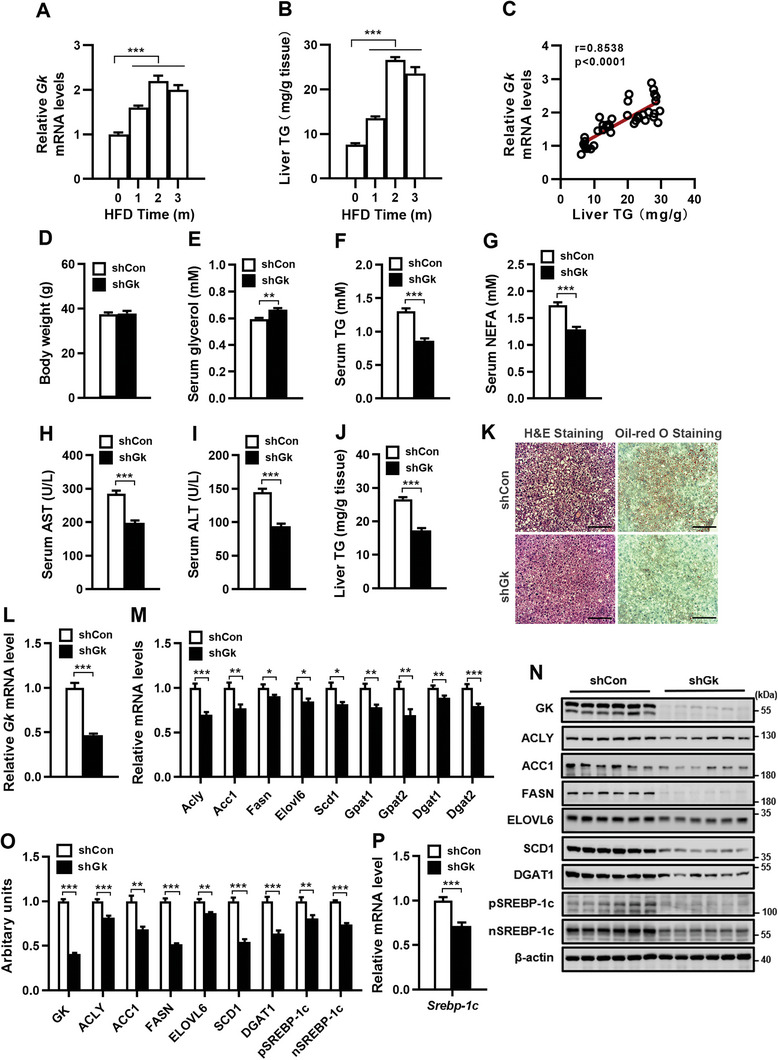
Knockdown of *Gk* in the liver attenuates HFD‐induced hepatic *Srebp‐1c* and lipogenic gene expression, hepatic steatosis, and hyperlipidemia. A–C) Hepatic Gk mRNA (A) and TG (B) levels in mice fed a high‐fat diet (HFD) for different periods of time, and Spearman correlation analysis between hepatic Gk mRNA and TG levels (C). D–P) Body weight (D), serum levels of glycerol (E), TG (F), NFFA (G), AST (H) and ALT (I), hepatic TG content (J), images of the liver tissue stained with H&E and Oil‐red O (K), as well as hepatic expression of *Gk*, lipogenic genes and *Srebp‐1c* at mRNA and protein levels (L‐P) in mice fed with HFD for 2 months followed by infection with adenoviruses expressing either Gk shRNA (shGk) or control sequence (shCon) for 2 weeks. n = 6‐8/group. Data are expressed as mean ± SEM. *p < 0.05, **p < 0.01, ***p < 0.001 (unpaired two‐tailed Student's *t* test). Scale bar: 100 µm. Acc, acetyl‐CoA carboxylase; Acly, ATP citrate lyase; ALT, alanine transaminase; AST, aspartate transaminase; Dgat, diacylglycerol acyltransferase; Elovl, elongase of very long chain fatty acids; Fasn, fatty acid synthase; Gk, glycerol kinase; Gpat, glycerol‐3‐phosphate acyltransferase; H&E, hematoxylin and eosin; NEFA, nonesterified fatty acids; Scd, stearoyl‐coenzyme A desaturase; Srebp‐1c, sterol regulatory element binding protein‐1c; nSREBP‐1c, nuclear form of SREBP‐1c; pSREBP‐1c, precursor of SREBP‐1c; TG, triglyceride.

The contribution of GK to NAFL was investigated in a mouse model of hepatic steatosis induced by HFD. There was a significant increase in hepatic GK expression at both mRNA and protein levels (Figure S1A,B, Supporting Information), body weight (Figure S1C, Supporting Information), serum levels of free fatty acids (Figure S1D, Supporting Information), and TG (Figure S1E, Supporting Information), as well as hepatic TG content (Figure S1F, Supporting Information) in mice fed an HFD for 2 months. Hepatic Gk mRNA levels and TG levels increased with time after HFD feeding (**Figure** **1**A,B). Spearman correlation analysis showed a positive association between hepatic Gk mRNA levels and hepatic TG levels (Figure 1C). As knockout of *Gk* leads to the death of mice within 3–4 days after birth,^[^ ^16^ ^]^ we examine the role of GK in hepatic steatosis by knocking down hepatic Gk in mice with HFD‐induced hepatic steatosis through adenovirus‐mediated RNA interference (RNAi). Infection of mice with adenoviruses expressing Gk shRNA significantly reduced GK expression in the liver, but not in other tissues (Figure S2, Supporting Information). Knockdown of hepatic *Gk* had no significant effect on body weight (Figure 1D). However, it resulted in a significant increase in serum glycerol levels (Figure 1E), and a remarkable decrease in serum levels of TG (Figure 1F), free fatty acids (Figure 1G), aspartate transaminase (AST) (Figure 1H), and alanine transaminase (ALT (Figure 1I), as well as liver TG content (Figure 1J). H&E staining and Oil Red O staining of liver tissues revealed a reduction in lipid deposition after *Gk* knockdown (Figure 1K). Knockdown of hepatic *Gk* in mice fed a HFD for 3 months also significantly lowered serum free fatty acids and TG levels, liver TG content, and alleviated hepatic lipid deposition (Figure S3, Supporting Information). Taken together, these findings suggest that the upregulation of hepatic *Gk* expression plays a critical role in the development of hepatic steatosis, hepatocyte damage, and hyperlipidemia induced by HFD consumption, possibly by regulating glycerol metabolism and TG synthesis.

We further examined the expression of genes involved in de novo lipogenesis and TG synthesis in the liver after Gk knockdown in mice with HFD‐induced hepatic steatosis, including *Acly*, *Acc1*, *Fasn*, *Elovl6*, *Scd1*, *Gpat*, and *Dgat*. Knocking down hepatic *Gk* resulted in a significant reduction of mRNA and protein levels of these genes in the liver of mice (Figure 1L–O). Furthermore, the expression of SREBP‐1c, a master regulator of these genes except for Dgat1/2, was significantly decreased at mRNA (Figure 1P) and protein levels (Figure 1N,O) in the liver. These results suggest that inhibition of hepatic Gk expression in HFD‐induced hepatic steatosis mice reduces de novo lipogenesis and TG synthesis by downregulating Srebp‐1c and Dgat1/2 expression.

Collectively, our results demonstrate that hepatic Gk expression is increased in HFD‐induced hepatic steatosis in mice. Increased hepatic Gk expression contributes to lipogenesis and lipid accumulation in the liver by catalyzing glycerol metabolism and regulating Srebp‐1c and Dgat1/2 gene expression.

### 2.2 Cholesterol and Fatty Acids Promote GK Expression in Hepatocytes

**Figure 2:**
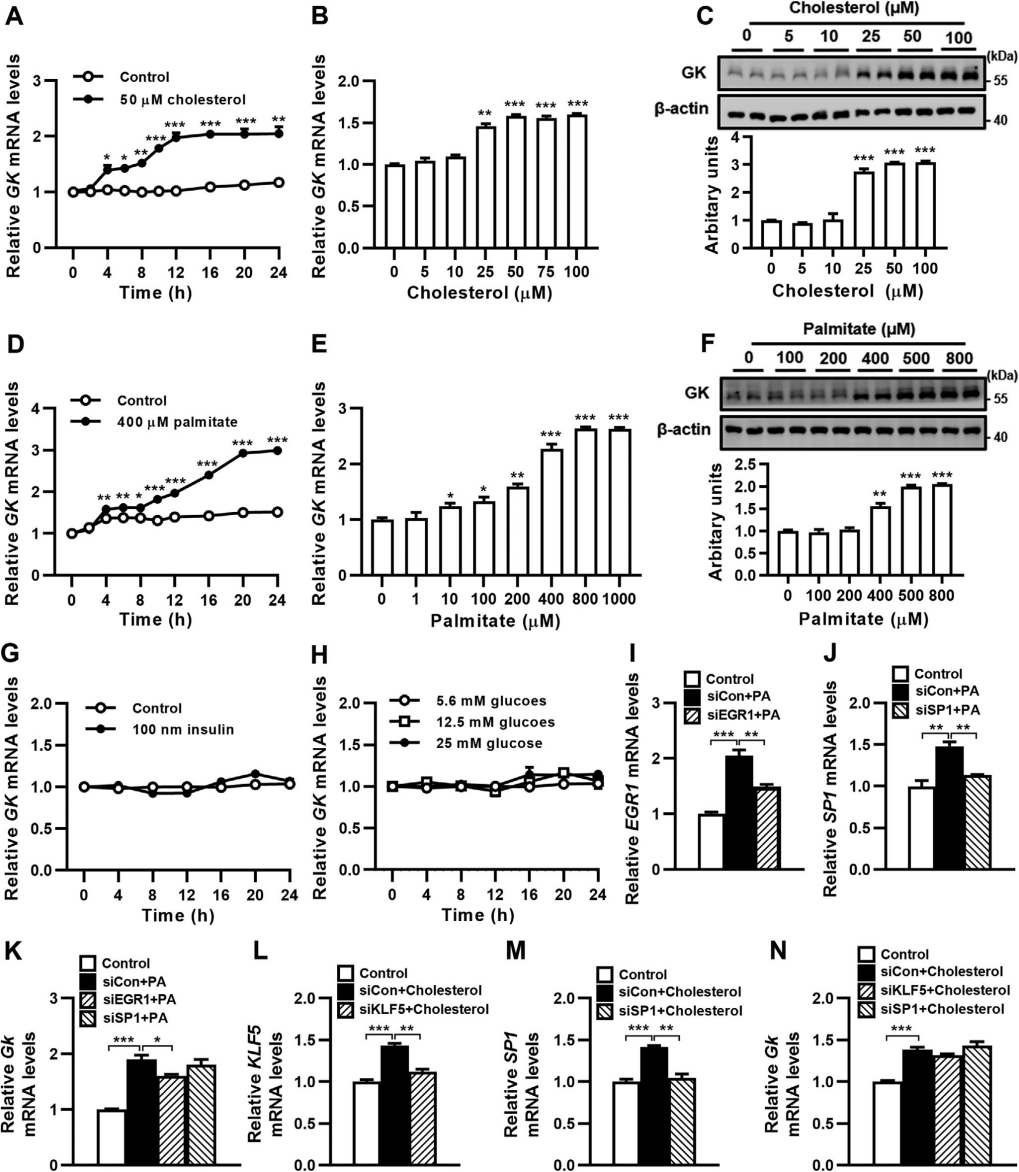
Cholesterol and fatty acids stimulate *GK* expression in hepatocytes. A,D,G,H) The *GK* mRNA levels in Huh7 cells stimulated with/without 50 µM cholesterol (A), 400 µM palmitate (B), 100 nM insulin (G), or different concentrations of glucose (H) for the indicated times. B,C,E,F) The mRNA (B,E) and protein (C, F) levels of GK in Huh7 cells treated with different concentrations of cholesterol or palmitate for 20 and 48 h, respectively. I‐K) The *EGR1*, *SP1*, and *GK* mRNA levels in Huh7 cells transfected with *EGR1* siRNA (siEGR1), *SP1* siRNA (siSP1) and stimulated with 400 µM palmitate (PA). L‐N) The *KLF5*, *SP1*, and *GK* mRNA levels in Huh7 cells transfected with *KLF5* siRNA (siEGR1), *SP1* siRNA (siSP1) and stimulated with 50 µM cholesterol. *n* = 3. Data are expressed as mean ± SEM. *p < 0.05, **p < 0.01, ***p < 0.001, compared with cells treated with control medium at the corresponding time points (unpaired two‐tailed Student's *t* test). GK, glycerol kinase; EGR1, early growth response 1; SP1, Sp1 transcription factor; KLF5, Krüppel‐like factor 5.

We investigated the factors that influence hepatic *Gk* expression in HFD‐fed mice. Our previous study found that mice on a HFD experienced a rise in blood cholesterol and free fatty acid levels after 1 and 2 weeks, respectively, and an increase in blood glucose and insulin levels after 1 month.^[^ ^17^ ^]^ Therefore, the impact of cholesterol, fatty acids, glucose, and insulin on GK expression in the Huh7 hepatocyte cell line was investigated. We observed that 50 µM cholesterol stimulated *GK* mRNA expression in Huh7 cells in a time‐dependent manner (**Figure** **2**A). Cholesterol at 25–100 µM significantly increased GK expression at both the mRNA and protein levels (Figure 2B,C). We found that 400 µM palmitate also promoted *GK* mRNA expression in a time‐dependent fashion (Figure 2D). Palmitate induced GK mRNA and protein expression in a dose‐dependent manner (Figure 2E,F). However, both 100 nM insulin and 5.6–25 mM glucose had no significant impact on *GK* mRNA expression (Figure 2G,H). Considering that the levels of blood cholesterol and free fatty acids were elevated prior to the observed increase in hepatic Gk expression among HFD‐fed mice, these results suggest that cholesterol and fatty acids play a role in the upregulation of hepatic Gk expression in HFD‐fed mice.

To investigate how cholesterol and palmitate regulate GK expression in hepatocytes, we predicted the binding sequences of transcription factors in the promoter region of the human GK gene (http://genome.ucsc.edu/index.html) using the JASPAR database (http://jaspar.genereg.net) and found that there are binding sites for Zinc Finger Protein 384 (ZNF384), ZNF148, Early Growth Response 1 (EGR1), SP1‐SP4, SP9, Krüppel‐like factor 5 (KLF5), KLF15 and KLF16. Palmitate has been reported to upregulate ERG1 and SP1 expression^[^ ^18^, ^19^ ^]^ and inhibit ZNF148, KLF15, and KLF16 expression,^[^ ^20^, ^21^, ^22^ ^]^ whereas cholesterol has been reported to stimulate SP1 and KLF5 expression.^[^ ^23^, ^24^ ^]^ We tested whether ERG1, SP1, and KLF5 are involved in the expression of GK induced by palmitate and cholesterol. Our results showed that 400 µM palmitate significantly induced *ERG1* and *SP1* mRNA expression in Huh7 cells, and the knockdown of *ERG1* but not *SP1* significantly attenuated palmitate‐induced *GK* expression (Figure 2I–K). These results indicate that palmitate induces *GK* expression in hepatocytes through the upregulation of *ERG1*. We found that 50 µM cholesterol significantly induced *KLF5* and *SP1* expression in Huh7 cells, knockdown of *KLF5* had a tendency to decrease cholesterol‐induced *GK* expression but knockdown of *SP1* had no effect on cholesterol‐induced *GK* expression (Figure 2L–N). These results suggest that KLF5 and SP1 may not be involved in cholesterol‐induced GK expression in hepatocytes.

### 2.3 Hepatic Gk Expression is Increased and Positively Associated with SREBP‐1c Expression in NAFL Mouse Models

**Figure 3:**
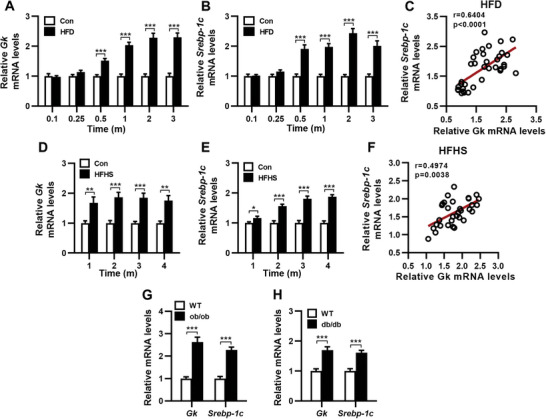
Expression of *Gk* and *Srebp‐1c* in the liver of diet‐induced and genetic mouse models of non‐alcoholic hepatic steatosis. A, B, D, E) The mRNA levels of hepatic glycerol kinase (*Gk*) and *Srebp‐1c* in mice fed with a high‐fat diet (HFD) (A,B), a high‐fat and high‐sucrose diet (HFHS) (D,E), or corresponding control diet (Con) for varying time durations. C, F) Spearman correlation analysis between the mRNA levels of *Gk* and *Srebp‐1c* in HFD‐fed mice (C) and HFHS‐fed mice (F). G, H) The mRNA levels of hepatic *Gk* and *Srebp‐1c* in *ob/ob* mice (G), *db/db* mice (H), and their corresponding wild‐type (WT) mice. A‐B: *n* = 6‐8/group; D‐E: *n* = 8/group; G: *n* = 6/group; H: *n* = 5/group. Data are expressed as mean ± SEM. *p < 0.05, **p < 0.01, ***p < 0.001 (unpaired two‐tailed Student's *t* test). m, month.

Since knockdown of hepatic Gk in mice with HFD‐induced hepatic steatosis significantly reduced the expression of Srebp‐1c and its target lipogenic genes and TG synthesis genes (Figure 1L–P), we investigated the relationship between the expression of hepatic Gk and Srebp‐1c in mouse models of NAFL induced by either diet or leptin/leptin receptor gene mutation. In mice fed HFD for different durations, hepatic *Gk* mRNA levels tended to increase after 1 week of feeding and significantly rose after 2 weeks (**Figure** **3**A). The expression of hepatic *Srebp‐1c* mRNA increased in a pattern similar to that of Gk (Figure 3B). Spearman correlation analysis showed that *Gk* expression was positively associated with *SREBP‐1c* expression (Figure 3C). In mice fed a high‐fat and high‐sucrose diet (HFHS) for varying periods, hepatic *Srebp‐1c* mRNA levels increased along with elevated *Gk* mRNA levels (Figure 3D,E). Gk expression was also positively associated with *SREBP‐1c* expression (Figure 3F). In the livers of *ob/ob* and *db/db* mice, both *Gk* and *Srebp‐1c* mRNA levels were significantly elevated (Figure 3G,H). These results suggest that the increase in hepatic Gk expression occurs before the development of hepatic steatosis and may contribute to the increase of hepatic Srebp‐1c expression in mice.

### 2.4 Enhanced Hepatic Gk Expression Promotes TG Synthesis through SREBP‐1c‐ Mediated de novo Lipogenesis

**Figure 4:**
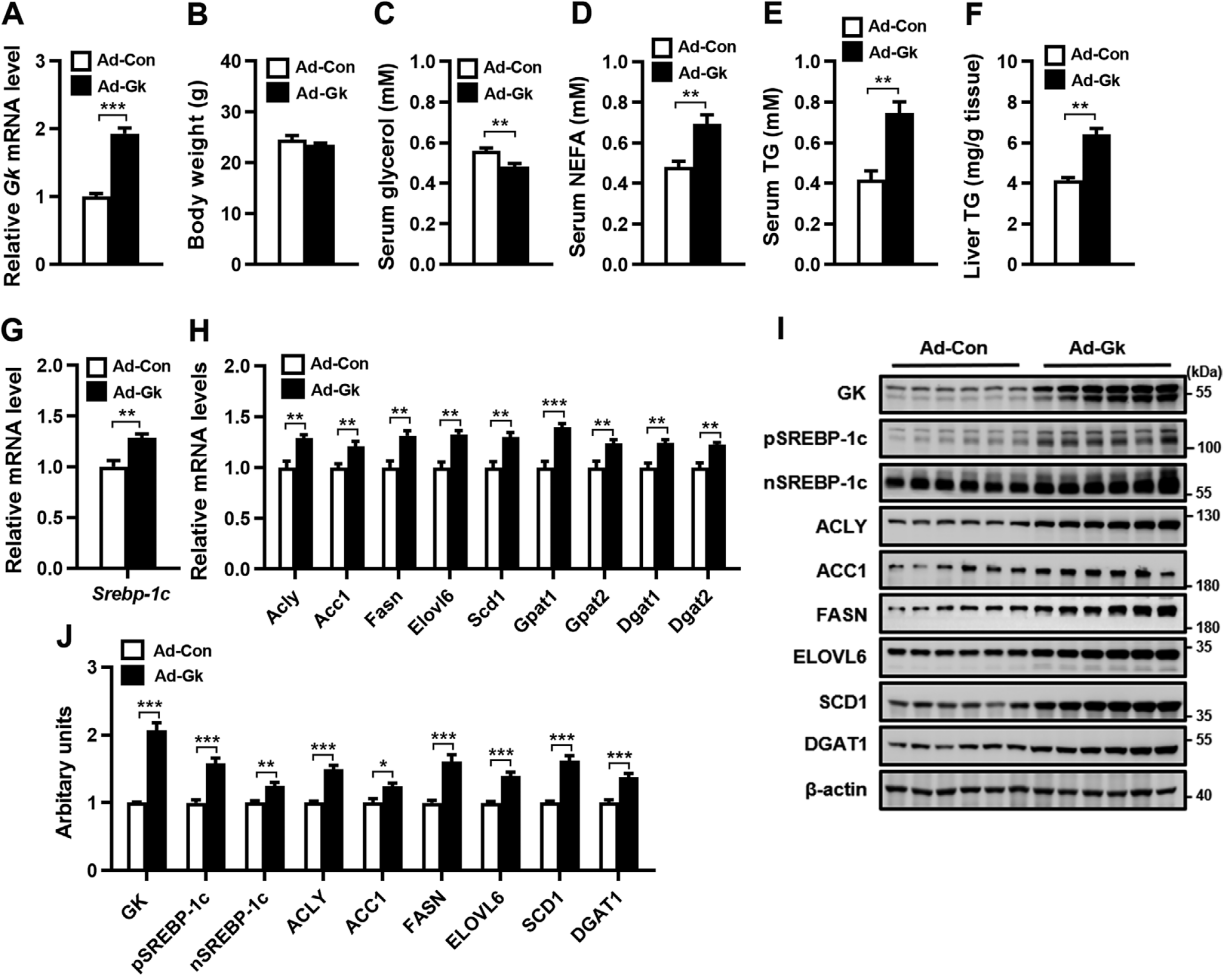
Enhanced Gk expression in mouse liver upregulates the expression of Srebp‐1c and its target lipogenic genes. The hepatic *Gk* mRNA (A), body weight (B), serum levels of glycerol (C), NEFA (D) and TG (E), liver TG content (F), as well as the mRNA and protein levels of hepatic *Srebp‐1c* and lipogenic genes (G–J) in mice infected with adenoviruses expressing Gk (Ad‐Gk) or carrying control vector (Ad‐Con). *n* = 6/group. Data are expressed as mean ± SEM. *p < 0.05, **p < 0.01, ***p < 0.001 (unpaired two‐tailed Student's *t* test). m, month; the other abbreviations in this figure are the same as in Figure 1.

We then investigated whether an increase in hepatic GK could upregulate the expression of *Srebp‐1c* and its target lipogenic genes by infecting mice with adenoviruses expressing Gk (Ad‐Gk) or a control vector (Ad‐Con). Ad‐Gk infection significantly increased hepatic expression of Gk mRNA and protein in mice compared to Ad‐Con infection (**Figure** **4**A,I,J). The infection of Ad‐GK had no significant impact on body weight (Figure 4B). However, it led to a significant decrease in serum glycerol levels (Figure 4C) and a significant increase in serum levels of free fatty acids (Figure 4D), TG (Figure 4E), and liver TG (Figure 4F). Furthermore, administration of Ad‐Gk to the mice resulted in the amplification of hepatic *Srebp‐1c* mRNA expression, along with the precursor and nuclear forms of SREBP‐1c protein (Figure 4G,I,J). In Ad‐Gk infected mice, both mRNA and protein levels of lipogenic genes downstream of *Srebp‐1c* and TG synthesis genes DGAT1/2 were upregulated (Figure 4H–J). As GK participates in TG synthesis by converting glycerol to glycerol‐3‐phosphate in the liver, these findings indicate that the increase in hepatic Gk expression not only regulates TG synthesis by regulating glycerol metabolism but also plays a critical role in de novo lipogenesis and TG synthesis by upregulating the expression of SREBP‐1c and its target lipogenic genes as well as TG synthesis genes.

**Figure 5:**
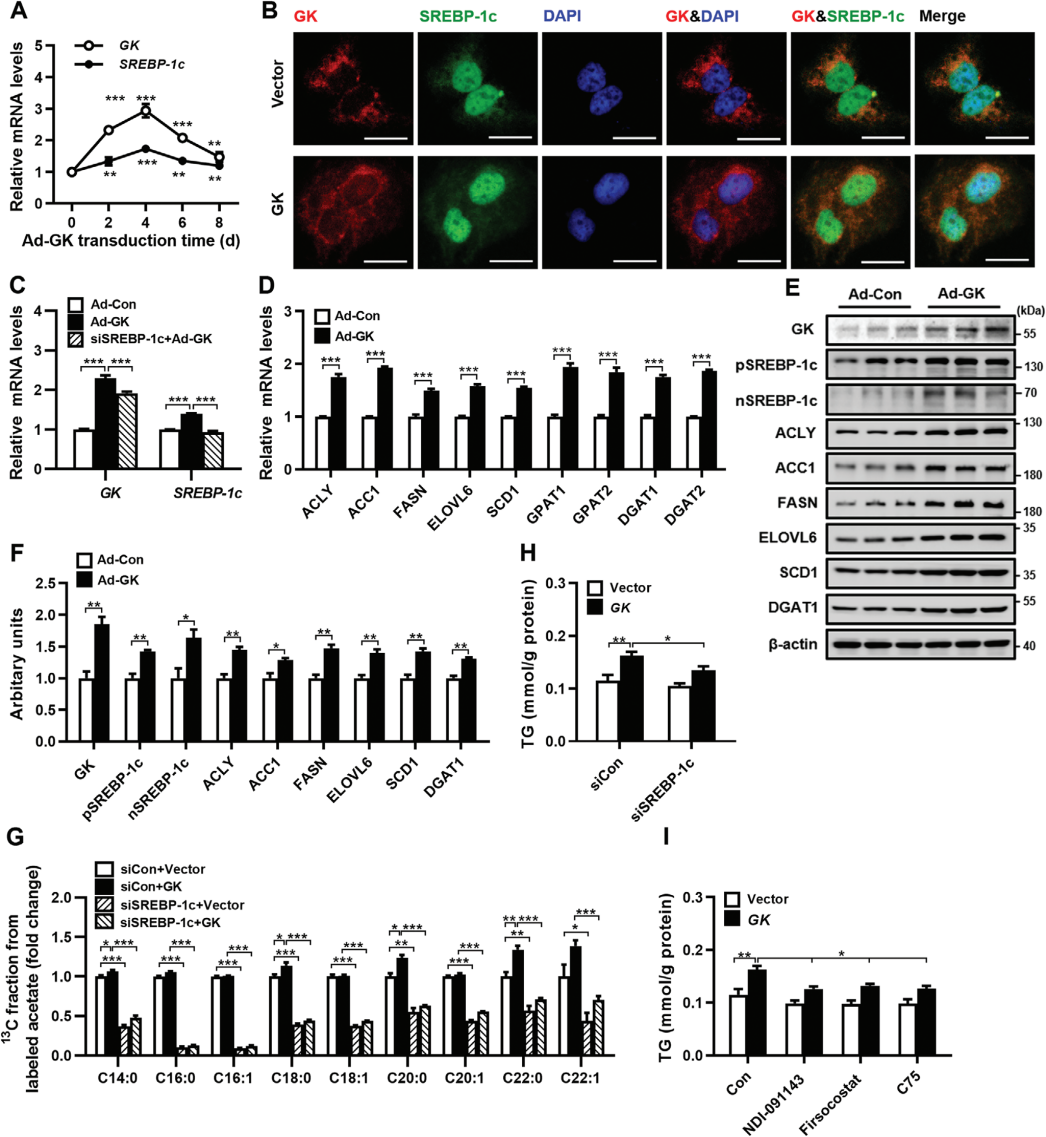
Overexpression of *GK* in hepatocytes promotes TG synthesis through upregulation of *SREBP‐1c* and its target lipogenic gene expression. A) The mRNA levels of *GK* and *SREBP‐1c* in Huh7 cells were transduced with adenoviruses expressing GK (Ad‐GK) for different time periods. B) Immunofluorescence staining of GK and SREBP‐1c in Huh7 cells transfected with GK expression plasmid (GK) or control vector. C) The mRNA levels of *GK* and *SREBP‐1c* in Huh7 cells infected with Ad‐GK or adenoviruses carrying control vector (Ad‐Con), or transfected with siRNA against SREBP‐1c (si*SREBP‐1c*) followed by infection with Ad‐GK. D–F) The mRNA (D) and protein (E,F) levels of lipogenic genes in Huh7 cells transduced with Ad‐Con or Ad‐*GK*. G) Metabolic flux analysis of de novo fatty acid synthesis by using [^13^C]‐acetate in HepG2 cells transfected with control siRNA (siCon) or siSREBP‐1c followed by transfection with GK expression plasmid (GK) or control vector. H) Intracellular TG levels in HepG2 cells transfected with control siRNA (siCon) or siSREBP‐1c followed by transfection with GK expression plasmid (GK) or control vector. I) Intracellular TG levels in HepG2 cells treated with ACLY inhibitor NDI‐091143, ACC inhibitor firsocostat, or FASN inhibitor C75 followed by transfection with *GK* expression plasmid (GK) or control vector. *n* = 3. Data are expressed as mean ± SEM. *p < 0.05, **p < 0.01, ***p < 0.001, compared to Huh7 cells without Ad‐GK transfection (unpaired two‐tailed Student's *t*‐test). Abbreviations in this figure are the same as in Figure 1.

We further investigated whether increased GK expression in hepatocytes could stimulate TG synthesis by upregulating SREBP‐1c expression and subsequent lipogenic gene expression. GK was overexpressed in Huh7 cells by infecting the cells with Ad‐GK. Following Ad‐*GK* infection, *GK* mRNA levels peaked on day 4 and then gradually reduced. The *SREBP‐1c* mRNA expression was elevated according to the pattern of *GK* mRNA (**Figure** **5**A). Immunofluorescence staining of Huh7 cells showed that GK was mainly localized in the cytoplasm, SREBP‐1c was present in both the cytoplasm and the nucleus, and GK co‐localized with SREBP‐1c in the cytoplasm. Overexpression of GK in Huh7 cells by transfecting the cells with GK expression plasmid increased GK and SREBP‐1c in the nucleus, and GK co‐localized with SREBP‐1c in the cytoplasm and nucleus (Figure 5B). Knockdown of *SREBP‐1c* by RNAi abolished GK‐induced *SREBP‐1c* expression (Figure 5C), indicating a direct effect of GK on *SREBP‐1c* expression in hepatocytes. Overexpression of *GK* in Huh7 cells not only stimulated *SREBP‐1c* expression (Figure 5A–C,E,F), but also upregulated the expression of lipogenic genes downstream of SREBP‐1c and TG synthesis genes *DGAT1/2* at both mRNA and protein levels (Figure 5D–F). To investigate the effect of GK on de novo fatty acid synthesis in hepatocytes, we performed metabolic flux analysis by incubating HepG2 cells with [^13^C]‐acetate. The results showed that overexpression of GK significantly increased the incorporation of ^13^C into C14:0, C18:0, C20:0 and C22:0 fatty acids. Knockdown of *SREBP‐1c* significantly reduced basal ^13^C incorporation and reversed GK‐induced ^13^C incorporation into these fatty acids (Figure 5G). These results indicate that GK promotes de novo lipogenesis via SREBP‐1c. Further studies showed that overexpression of GK in HepG2 cells significantly increased intracellular TG levels (Figure 5H). To determine whether GK promotes TG synthesis through SREBP‐1c and de novo lipogenesis in hepatocytes, we transfected HepG2 cells with siRNA against *SREBP‐1c* or treated the cells with ACLY inhibitor NDI‐091143, ACC inhibitor firsocostat, or FASN inhibitor C75. While knockdown of *SREBP‐1c* or inhibition of ACLY, FASN or ACC had no significant effect on basal intracellular TG levels, these treatments significantly inhibited the increase in intracellular TG induced by GK (Figure 5H,I). These results demonstrate that increasing GK levels in hepatocytes stimulate TG synthesis in part by increasing *SREBP‐1c* and lipogenic gene expression, leading to de novo lipogenesis and TG synthesis.

### 2.5 GK Promotes *SREBP‐1c* Transcription by Binding to the Promoter of SREBP‐1c Gene and Interaction with SREBP‐1c Protein

**Figure 6:**
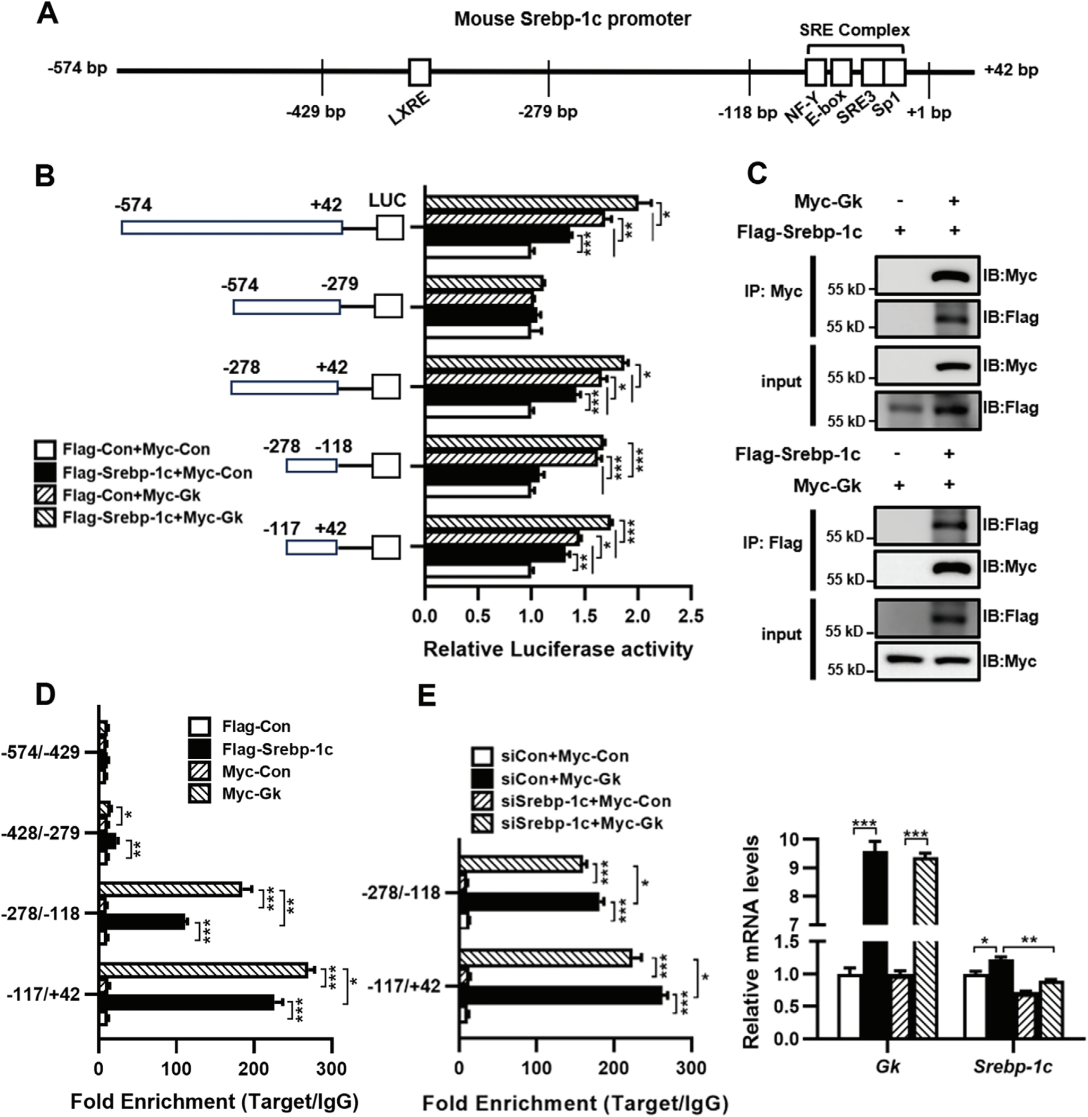
Mouse GK enhances *Srebp‐1c* transcription by binding to the promoter of the Srebp‐1c gene and the SREBP‐1c protein. A) Schematic representation of the mouse *Srebp‐1c* gene promoter with the major cis‐acting elements. B) Schematic illustration of the mouse Srebp‐1c promoter‐luciferase reporter constructs. Luciferase activity in HEK 293T cells co‐transfected with the indicated promoter‐luciferase reporter constructs and either control vectors or expression plasmids for GK, SREBP‐1c, or both. C) Co‐IP of Myc‐GK and Flag‐SREBP‐1c in HEK293T cells. D–E) ChIP assay of Myc‐GK and Flag‐SREBP‐1c binding to the promoter regions of the mouse Srebp‐1c gene in AML12 cells without/with Srebp‐1c knockdown by RNA interference. *n* = 3. Data are expressed as mean ± SEM. *p < 0.05, **p < 0.01, ***p < 0.001 (unpaired two‐tailed Student's *t* test). GK, glycerol kinase; Srebp‐1c, sterol regulatory element binding protein‐1c.

To investigate the molecular mechanisms underlying the regulation of Srebp‐1c mRNA expression by GK, luciferase reporter constructs containing various regions of the mouse *Srebp‐1c* promoter (**Figure** **6**A,B) were generated. Luciferase activity in HEK 293T cells was measured after transfection with these constructs and plasmids expressing Gk or Srebp‐1c. As shown in Figure 6B, co‐transfection of HEK 293T cells with plasmids expressing either Gk or Srebp‐1c with (−574/+42)‐Luc significantly increased luciferase activity. Co‐transfection of the cells with expression plasmids for Gk, Srebp‐1c, and (−574/+42)‐Luc further elevated luciferase activity. However, co‐transfection of the cells with plasmids expressing Gk or Srebp‐1c with (−574/−279)‐Luc had no effect on luciferase activity. These results indicate that GK stimulates *Srebp‐1c* transcription and enhances SREBP‐1c‐induced *Srebp‐1c* transcription independently of the (−574/−279) region of the *Srebp‐1c* promoter. Luciferase reporter assays showed that co‐expression of Gk, Srebp‐1c, or both with (−278/+42)‐Luc resulted in similar outcomes as co‐expression of Gk, Srebp‐1c, or both with (−574/+42)‐Luc, supporting that GK and SREBP‐1c activate *Srebp‐1c* transcription by binding to the (−278/+42) region of the *Srebp‐1c* promoter. To determine the binding site of GK in this region, we constructed (−278/−118)‐Luc and (−117/+42)‐Luc. The luciferase reporter assays showed a significant increase in luciferase activity when Gk was co‐expressed with either (−278/−118)‐Luc or (−117/+42)‐Luc. These results indicate that the (−117/+42) and (−278/−118) DNA sequences within the Srebp‐1c promoter are essential for GK to induce *Srebp‐1c* transcription. Co‐expression of Srebp‐1c with (−117/+42)‐Luc, rather than (−278/−118)‐Luc, significantly increased luciferase activity. This result is consistent with a previous report that the (−117/+42) region contains an SRE complex that can be bound by SREBP‐1c to induce *Srebp‐1c* transcription.^[^ ^25^ ^]^ Co‐expression of Gk and Srebp‐1c with (−117/+42)‐Luc resulted in higher luciferase activity than co‐expression of Srebp‐1c with (−117/+42)‐Luc, indicating that GK enhances SREBP‐1c‐induced *Srebp‐1c* transcription by binding to this region or binding to the SREBP‐1c protein. To investigate whether GK could bind to SREBP‐1c, plasmids expressing Gk or Srebp‐1c with tags were introduced into HEK 293T cells by co‐transfection. The Co‐IP assay showed that GK was co‐immunoprecipitated with SREBP‐1c, indicating a direct binding between GK and SREBP‐1c (Figure 6C). ChIP assays were performed to check whether GK could directly bind to the *Srebp‐1c* promoter by transfecting mouse AML12 hepatocytes with the plasmids expressing tagged GK or SREBP‐1c. The results showed that both GK and SREBP‐1c could associated with the (−117/+42) and (−278/−118) DNA sequences within the *Srebp‐1c* promoter. GK exhibited more binding than SREBP‐1c (Figure 6D). Further study showed that the knockdown of endogenous *Srebp‐1c* significantly reduced the binding of GK to these regions (Figure 6E). Since the luciferase reporter assay showed that SREBP‐1c activates *Srebp‐1c* transcription by binding to the (−117/+42) region rather than the (−278/−118) region of the *Srebp‐1c* promoter (Figure 6B), these results indicate that GK promotes *Srebp‐1c* transcription by binding to the (−117/+42) and (−278/−118) regions of mouse *Srebp‐1c* promoter, as well as to the SREBP‐1c protein.

**Figure 7:**
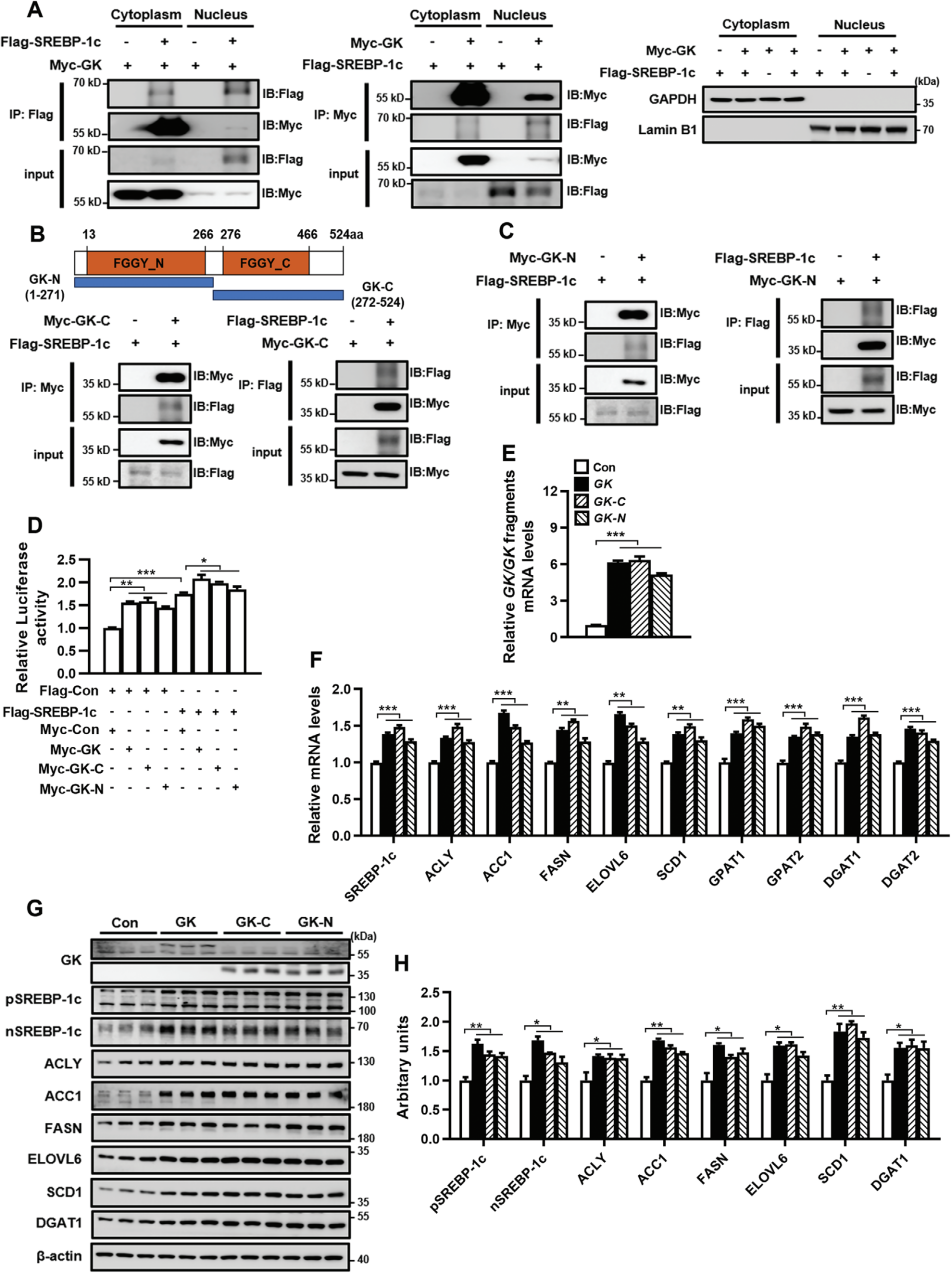
Human GK binds SREBP‐1c and induces *SREBP‐1c* gene transcription. A) Co‐IP of Myc‐GK and Flag‐SREBP‐1c in cytoplasm and nuclei of HEK‐293T cells. B,C) Co‐IP of Myc‐GK fragments (GK‐N, GK‐C) and Flag‐SREBP‐1c in HEK 293T cells. The domain structures of GK, GK‐N, and GK‐C are shown. D) Luciferase activity in HEK 293T cells co‐transfected with a human *SREBP‐1c* promoter‐luciferase reporter construct and the plasmids indicated. E–H) The expression of *GK*, *GK* fragments,*SREBP‐1c*, and its target lipogenic genes at mRNA (E, F) and protein (G, H) levels in Huh7 cells transfected with control vector or plasmid expressing either GK or GK fragments. *n* = 3. Data are expressed as mean ± SEM. *p < 0.05, **p < 0.01, ***p < 0.001 (unpaired two‐tailed Student's *t* test). GK‐C, C‐terminal fragment of GK; GK‐N, N‐terminal fragment of GK. The other abbreviations in this figure are the same as in Figure 1.

We further investigated whether human GK could interact with SREBP‐1c and promote its transcription. Co‐IP assay showed that human GK co‐immunoprecipitated with SREBP‐1c in whole cell lysate (Figure S4, Supporting Information), cytoplasm, and nucleus (**Figure** **7**A), indicating that GK binds to SREBP‐1c in both cytoplasm and nucleus. Using truncated GK that contains either the N‐terminal FGGY domain (GK‐N) or the C‐terminal FGGY domain (GK‐C), we observed that both GK‐N and GK‐C were able to interact with SREBP‐1c (Figure 7B,C). The luciferase reporter assay, using a luciferase reporter driven by human *SREBP‐1c* gene promoter, showed that both GK and SREBP‐1c significantly increased luciferase activity, GK or GK fragments further enhanced SREBP‐1c‐induced luciferase activity (Figure 7D). These results indicate that human GK not only promotes SREBP‐1c gene transcription but also enhances SREBP‐1c‐induced self‐gene transcription, similar to that of mouse GK. Further studies showed that overexpression of either GK or GK fragments in Huh7 cells significantly upregulated the expression of SREBP‐1c and its target lipogenic genes as well as TG synthesis genes DGAT1/2 at both mRNA and protein levels (Figure 7E–H). Collectively, these findings indicate that human GK positively regulates *SREBP‐1c* transcription and *DGAT1/2* expression in hepatocytes in a manner similar to that of mouse GK. The N‐ and C‐terminal fragments of GK were sufficient to trigger SREBP‐1c gene transcription by binding to the *SREBP‐1c* promoter and to enhance SREBP‐1c‐induced self‐gene transcription by binding to the SREBP‐1c protein.

### 2.6 GK Promotes *SREBP‐1c* Transcription Independently of its Enzyme Activity

**Figure 8:**
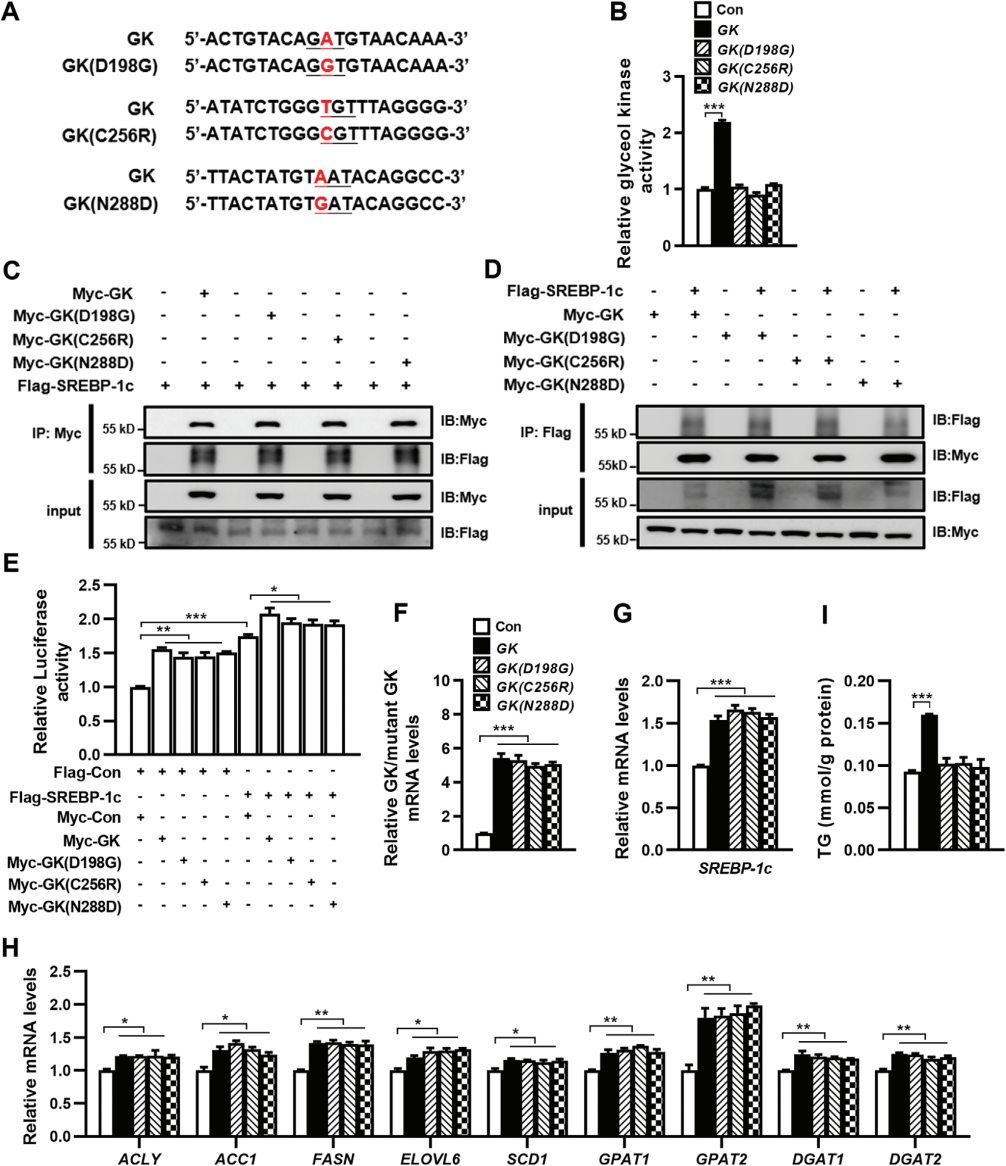
GK enhances *SREBP‐1c* expression independently of its kinase activity. A) The mRNA sequences for human wild type (WT) *GK* and *GK* with point mutation (593A→G: D198G, 766T→C: C256R, 862A→G: N288D). B) The GK activity in HEK 293T cells expressing WT GK, mutant GKs, or control vector (Con). C, D) Co‐IP of Myc‐GK/ Myc‐mutant GK and Flag‐SREBP‐1c in HEK 293T cells. E) Luciferase activity in HEK 293T cells co‐transfected with a human *SREBP‐1c* promoter‐luciferase reporter construct and the plasmids indicated. F–H) The expression of *GK*, mutant *GKs*, *SREBP‐1c*, and its target lipogenic genes as well as *DGAT1/2* at mRNA level in HepG2 cells transfected with a plasmid expressing GK, mutant GKs, or control vector. I) Intracellular TG levels in HepG2 cells transfected with WT GK, mutant GKs, or control vector (Con) for 36 h. *n* = 3. Data are expressed as mean ± SEM. *p < 0.05, **p < 0.01, ***p < 0.001 (unpaired two‐tailed Student's *t* test). Abbreviations in this figure are the same as in Figure 1.

We investigated whether the enzyme activity of GK is essential for its regulation of SREBP‐1c transcription by constructing plasmids expressing GK with point mutations found in patients with glycerol kinase deficiency^[^ ^26^ ^]^ (**Figure** **8**A). HEK 293T cells transfected with the wild‐type (WT) GK expression plasmid exhibited higher glycerol kinase activity compared to cells transfected with the control vector. In contrast, introducing mutant GKs into the cells did not affect enzyme activity (Figure 8B), confirming the lack of enzyme activity in these GK mutants. Each mutant GK was shown to interact with SREBP‐1c by Co‐IP analysis (Figure 8C,D). The luciferase reporter assay, which used the luciferase gene driven by the human SREBP‐1c gene promoter, showed that mutant GKs increased luciferase activity and further elevated the luciferase activity induced by SREBP‐1c (Figure 8E). These results indicate that mutant GKs, like WT GK, activate SREBP‐1c transcription by binding to the promoter of SREBP‐1c and to the SREBP‐1c protein. Further studies showed that overexpression of WT GK or mutant GKs in HepG2 cells significantly increased *SREBP‐1c* and its downstream lipogenic genes and *DGAT1/2* expression at mRNA level (Figure 8F–H). Overexpression of WT GK in HepG2 significantly increased intracellular TG levels, but overexpression of mutant GKs had no significant effect on intracellular TG levels (Figure 8I), indicating that although mutant GKs could promote de novo lipogenesis by upregulating *Srebp‐1c* expression, they could not catalyze glycerol to glycerol‐3‐phosphate to participate in TG synthesis due to the lack of glycerol kinase activity. Taken together, these findings indicate that GK promotes *SREBP‐1c* transcription independently of its enzyme activity.

### 2.7 Hepatic GK does not Regulate the Expression of *Srebp‐1c* Under Physiological Conditions in Mice

**Figure 9:**
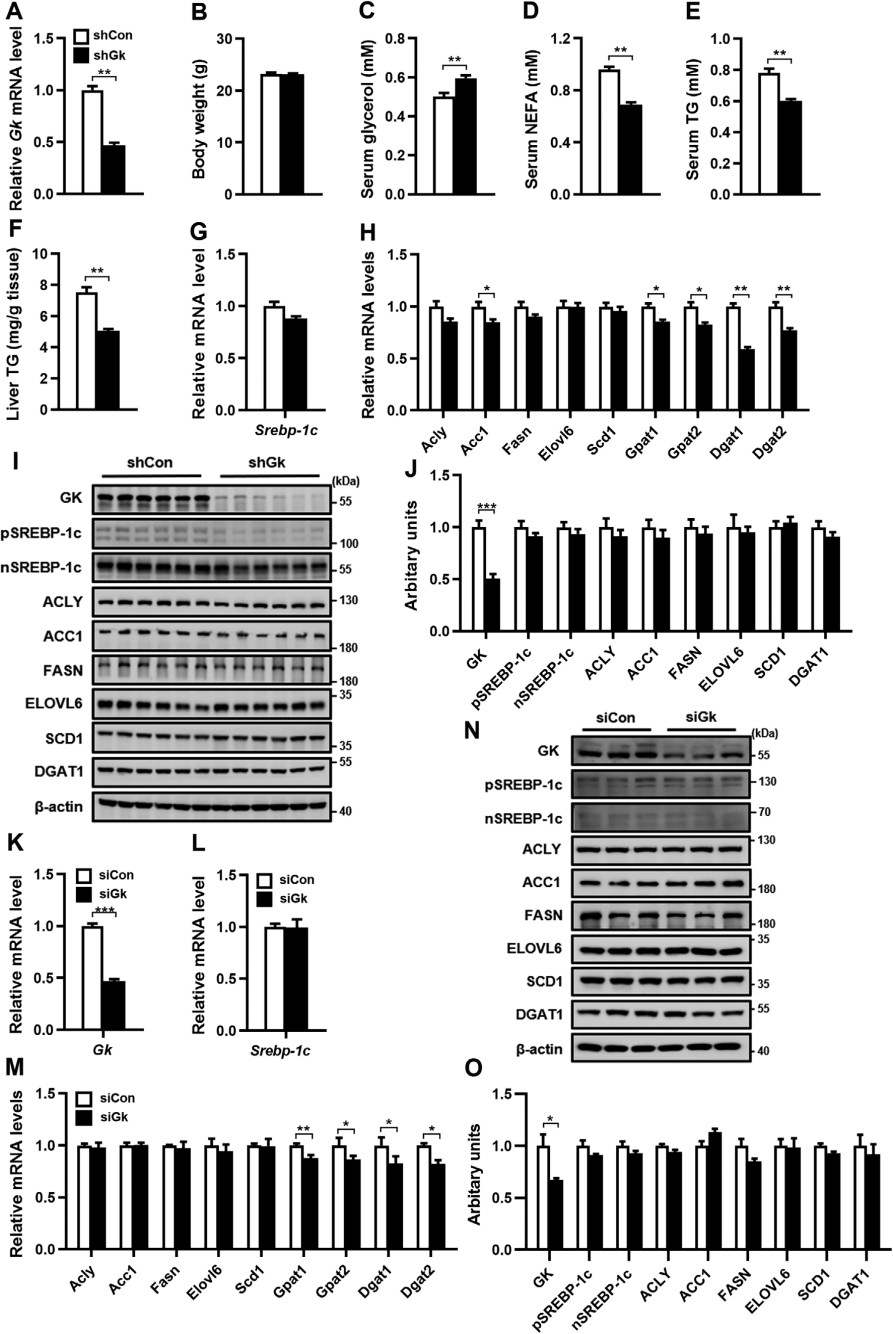
Knockdown of *Gk* in mouse liver or hepatocytes does not significantly affect the expression of *Srebp‐1c* and its target lipogenic genes. A–J) Hepatic *Gk* mRNA level (A), body weight (B), serum levels of glycerol (C), NEFA (D) and TG (E), liver TG content (F), as well as the expression of hepatic *Srebp‐1c*, lipogenic genes, and TG synthesis genes at mRNA and protein levels (G‐J) in mice infected with adenoviruses expressing Gk shRNA (shGK) or control sequence (shCon) for 2 weeks. *n* = 6/group. K–O) The mRNA levels of *GK*, *SREBP‐1c*, lipogenic genes, and TG synthesis genes in AML12 cells transfected with Gk siRNA (siGk) or control siRNA (siCon). *n* = 3. Data are expressed as mean ± SEM. *p < 0.05, **p < 0.01, ***p < 0.001 (unpaired two‐tailed Student's *t* test). Abbreviations in this figure are the same as in Figure 1.

To examine whether hepatic GK regulates *Srebp‐1c* expression under physiological conditions, mice on a normal chow were infected with adenoviruses expressing either Gk shRNA (shGk) or scrambled control shRNA (shCon). The infection of shGk resulted in a significant reduction of hepatic GK at both mRNA and protein levels compared to shCon infection (**Figure** **9**A,I,J), but had no significant effect on GK expression in other tissues (Figure S5, Supporting information). Knockdown of hepatic GK did not affect body weight (Figure 9B). However, it significantly increased serum glycerol levels (Figure 9C), and decreased serum levels of free fatty acids (Figure 9D), TG (Figure 9E), and liver TG (Figure 9F). The expression of Srebp‐1c at both mRNA and protein levels was not significantly affected by the knockdown of hepatic GK (Figure 9G,I,J). Among the analyzed target lipogenic genes of SREBP‐1c, knockdown of Gk resulted in a decrease of *Acc1and Gpat1/2* at the mRNA level but not at the protein level. Similarly, the knockdown of hepatic GK reduced DGAT1/2 at the mRNA level but not at the protein level (Figure 9H–J). These findings indicate that hepatic GK plays an important role in lipid homeostasis by regulating glycerol metabolism rather than *Srebp‐1c* expression under physiological conditions. Consistent with the results in mice, knockdown of GK in the normal mouse hepatocyte cell line AML12 by RNAi had no significant effect on the expression of SREBP‐1c and its target lipogenic genes ACLY, ACC1, FASN, ELOCL6 and SCD1 at both mRNA and protein levels, and reduced Gpat1/2 and Dgat1/2 at mRNA but not at protein levels (Figure 9K–O). These results indicate that the basal level of GK in hepatocytes does not regulate *SREBP‐1c* expression.

## 3 Discussion

In the present study, we found that hepatic Gk and Srebp‐1c expression increased in mice with NAFL induced by both diet and gene mutation. Cholesterol and fatty acids stimulated GK expression in hepatocytes. Knockdown of hepatic GK in HFD‐fed mice reduced the expression of SREBP‐1c and its target lipogenic genes as well as TG synthesis genes, de novo lipogenesis, TG synthesis, and TG deposition in the liver, and attenuated hepatocyte damage. Overexpression of GK in the hepatocytes in mice or in culture resulted in opposite outcomes. Mechanistic studies revealed that GK promoted *SREBP‐1c* gene transcription by binding to its promoter and to the SREBP‐1c protein. The elevation of hepatic GK‐induced *SREBP‐1c* transcription is independent of its enzyme activity. The basal level of hepatic GK had no effect on *SREBP‐1c* transcription under physiological conditions.

In diet‐induced and genetic mouse models of NAFL, we observed an increase in hepatic Gk expression. After HFD feeding, the increase in hepatic *Gk* mRNA was positively associated with hepatic TG levels. There was also a positive association between hepatic Gk mRNA levels and serum TG levels in these mice (Figure S6, Supporting Information). These results strongly suggest that elevation of hepatic GK plays a crucial role in HFD‐induced hepatic TG accumulation and hypertriglyceridemia. We demonstrated that GK expression in hepatocytes was stimulated by cholesterol and palmitate, but not by glucose and insulin. Palmitate stimulated GK expression in hepatocytes by upregulating the expression of *ERG1*. However, the transcription factors involved in cholesterol‐induced GK expression require further investigation. It has been reported that circulating levels of cholesterol, fatty acids, glucose, and insulin increased sequentially in mice after HFD feeding.^[^ ^17^ ^]^ HFHS‐fed mice have a significant increase in blood cholesterol levels.^[^ ^27^ ^]^ Ob/ob mice and db/db mice have higher blood cholesterol and fatty acid levels compared to control mice.^[^ ^28^ ^]^ Therefore, the increased expression of hepatic Gk in these mouse models of NAFL may result from the elevation of circulating cholesterol and fatty acids.

GK converts glycerol to glycerol 3‐phosphate, which can be re‐esterified to TG. The contribution of elevated hepatic GK to NAFL was investigated by knockdown of *Gk* in the livers of mice through adenovirus‐mediated RNAi. Our study showed that knockdown of hepatic *Gk* in mice with HFD‐induced hepatic steatosis significantly increased serum levels of glycerol, reduced the expression of *Srebp‐1c* and its target lipogenic genes as well as TG synthesis genes in the liver, lowered serum lipid levels, and decreased hepatic TG deposition. The overexpression of hepatic Gk in mice resulted in opposite outcomes. These findings indicate that the increase in hepatic Gk plays a critical role in HFD‐induced hepatic steatosis in mice, by promoting glycerol metabolism and upregulating *Srebp‐1* and *Dgat1/2* expression. Additionally, we found that the increase in hepatic *Srebp‐1c* expression was also present in other NAFL mouse models, including HFHS‐fed mice and mice with mutations in the leptin/leptin receptor gene. The increase in *Srebp‐1c* expression was positively associated with an increase in *Gk* expression. These results indicate that upregulation of *Gk* expression may be a common occurrence in NAFL and is a positive regulator of *Srebp‐1c* expression. By overexpressing *Gk* in the liver of mice and in hepatocytes in vitro, and inhibiting either *SREBP‐1c* expression or enzymes involved in de novo lipogenesis and TG synthesis, we demonstrated that elevation of GK stimulated the expression of *Srebp‐1c* and its target genes as well as TG synthesis genes *DGAT1/2* involved in fatty acid and TG synthesis, resulting in increased fatty acid synthesis and TG levels in hepatocytes. Consistent with our results, overexpression of human GK in a rat hepatoma cell line resulted in significantly increased *SREBP‐1* mRNA and lipid accumulation in the cells.^[^ ^29^ ^]^ It has been reported that upregulation of DGAT2 expression may promote lipogenesis by regulating cleavage of SREBP‐1c precursor protein.^[^ ^30^ ^]^ Therefore, GK may also promote lipogenesis and TG synthesis by regulating SREBP‐1c processing through DGAT2 in hepatocytes.

SREBP‐1c is crucial in maintaining the transcription of lipogenic genes that encode enzymes for fatty acid and TG synthesis in the liver under physiological conditions.^[^ ^31^ ^]^ The transcription of the SREBP‐1c gene is regulated by fasting/feeding,^[^ ^32^ ^]^ a high cholesterol‐diet,^[^ ^33^ ^]^ and insulin,^[^ ^34^ ^]^ Insulin induces *Srebp‐1c* transcription by activating the liver X receptor responsive elements and SRE in the promoter of the *SREBP‐1c* gene.^[^ ^35^, ^36^ ^]^ It also enhances the processing of the SREBP‐1c precursor protein.^[^ ^37^ ^]^ In mouse models of NAFL with insulin resistance, hepatic SREBP1c activity is increased and plays an essential role in hepatic lipid accumulation,^[^ ^38^ ^]^ suggesting that elevated insulin levels stimulate *Srebp‐1c* transcription and SREBP‐1c precursor protein processing. However, the other stimulators of *SREBP‐1c* transcription in NAFL are currently unknown. GK has been reported to upregulate the expression of hepatic SREBP‐1c in normal and diabetic mice through repressing nuclear receptor subfamily 4 group A1, a negative regulator of SREBP‐1c.^[^ ^39^ ^]^ Our study identified GK as a novel positive regulator of SREBP‐1c transcription in NAFL. Immunofluorescence staining results showed that GK protein was mainly localized in the cytoplasm of hepatocytes. After overexpression of GK in hepatocytes, GK was present in both the cytoplasm and nucleus of hepatocytes, which is consistent with the results reported previously.^[^ ^40^, ^41^ ^]^ Furthermore, GK co‐localized with SREBP‐1c in the cytoplasm and nucleus of GK‐overexpressing cells. Immunoprecipitation results confirmed that GK bound to SREBP‐1c in both cytoplasm and nucleus. The mechanism of GK entry into the nucleus is currently unknown. Protein trafficking from the cytoplasm to the nucleus is mainly mediated by the nuclear pore complex (NPC). The nuclear localization sequence in the cargo protein is recognized by the nuclear transporters, which interact with nucleoporins to help the cargo protein enter the nucleus through the NPC.^[^ ^42^ ^]^ We analyzed the sequence of GK protein with NLSdb database (https://rostlab.org/services/nlsdb/) and did not find any currently known nuclear localization sequences.^[^ ^43^ ^]^ It has been reported that some proteins enter the nucleus through the NPC in a manner that does not depend on nuclear localization sequences, such as β‐catenin and MeCP2.^[^ ^44^, ^45^ ^]^ We hypothesize that GK may enter the nucleus independently of the nuclear localization sequence, but the exact mechanism requires further investigation. Through luciferase reporter assay, ChIP assay, and Co‐IP assay, we demonstrated that GK stimulates SREBP‐1c transcription by binding to the −278/−118 and −117/+42 regions of the mouse *Srebp‐1c* gene promoter, and enhances SREBP‐1c‐induced self‐gene transcription by binding to the SREBP‐1c protein. Additionally, we found that GK‐N, GK‐C, and mutant GKs were able to bind to the SREBP‐1c protein and stimulate *SREBP‐1c* transcription in a manner similar to that of WT GK. Since GK‐N contains an FGGY N‐terminal domain primarily involved in substrate binding, GK‐C contains an FGGY C‐terminal domain mainly responsible for ATP binding, mutant GK has no enzyme activity, these findings indicate that GK stimulates *SREBP‐1c* transcription independently of its enzyme activity.

We investigated whether GK regulates *SREBP‐1c* transcription under physiological conditions and contributes to lipid homeostasis. Our results showed that the knockdown of hepatic *Gk* in mice resulted in a significant increase in serum glycerol levels, and a reduction in liver and blood TG levels, but had no significant effect on the expression of hepatic SREBP‐1c and its downstream lipogenic proteins. Knockdown of *Gk* in the normal mouse hepatic cell line also did not affect the expression of SREBP‐1c and lipogenic proteins. These results indicate that hepatic GK plays an important role in lipid homeostasis under physiological conditions by regulating TG synthesis by catalyzing glycerol metabolism rather than by regulating *SREBP‐1* transcription. Since immunofluorescence staining showed that GK was mainly located in the cytoplasm and co‐localized with SREBP‐1c in the cytoplasm of hepatocytes under normal conditions, the low level of GK in the nucleus or the lack of factors that regulate GK nuclear translocation may make GK insufficient to regulate *SREBP‐1c* transcription.

**Figure 10:**
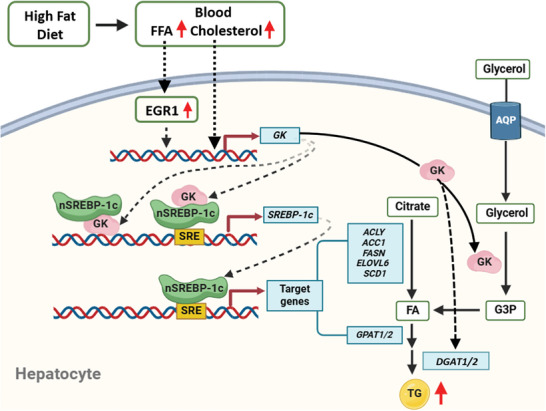
The schematic model of the contribution of hepatic GK to HFD‐induced non‐alcoholic hepatic steatosis. The expression of GK in the liver of mice fed a HFD is upregulated by FFA and cholesterol. The elevated GK catalyzes glycerol to G3P and stimulates *DGAT1/2* expression, thereby promoting TG synthesis. In addition, increased GK stimulates the transcription of SREBP‐1c by binding to the promoter of the SREBP‐1c gene, and enhances SREBP‐1c‐induced self‐gene transcription by binding to the SREBP‐1c protein. SREBP‐1c promotes the expression of genes involved in de novo lipogenesis and TG synthesis, resulting in the accumulation of TG in hepatocytes. AQP, aquaporin; FA, fatty acid; FFA, free fatty acid; G3P, glycerol‐3‐phosphate; SRE, sterol‐regulatory element; EGR1, early growth response 1. The other abbreviations in this figure are the same as in Figure 1.

In summary, this study demonstrates that hepatic GK plays a critical role in maintaining lipid homeostasis under physiological conditions and contributes to the development of NAFL. Hepatic Gk is upregulated by cholesterol and fatty acids during the development of NAFL induced by a HFD. We identify GK as a novel positive regulator of SREBP‐1c transcription in the development of NAFL. It contributes to de novo lipogenesis and TG synthesis in the liver by catalyzing glycerol metabolism and upregulating *SREBP‐1c* transcription and *DGAT1/2* expression (**Figure** **10**). GK may be a potential therapeutic target for NAFL.

## 4 Experimental Section

### Animals and Treatment

Male C57BL/6J mice were obtained from Shanghai Laboratory Animal Co. (Shanghai, China). *db/db* mice and *ob/ob* mice were purchased from the Shanghai Model Organisms Center (Shanghai, China). All mice were maintained and used in accordance with the guidelines and under the approval of the Institutional Animal Care and Use Committee of the Shanghai Institute of Nutrition and Health, Chinese Academy of Sciences. Eight‐week‐old C57BL/6J mice were fed with a control diet (D12450J, Research Diets), a high‐fat diet (D12492, Research Diets), or a high‐fat and high‐sucrose diet (D12327, Research Diets). The mice were euthanized with isoflurane at the indicated time points, and liver tissues and blood serum were collected and stored at −80 °C for further analysis. To overexpress *Gk* in the liver of mice on a control diet, eight‐week‐old mice were injected with adenoviruses expressing mouse *Gk* (Ad‐Gk) or vector (Ad‐Con). To knock down hepatic *Gk* in normal mice or HFD‐induced hepatic steatosis mice, the mice were injected with adenoviruses expressing short hairpin RNA (sh*Gk*) or control scrambled shRNA (shCon). The adenoviruses were dissolved in 0.2 mL PBS and administered at a dose of 1 × 10^9^ plaque‐forming units/mouse by tail vein injection. The mice were euthanized 2 weeks after injection, and blood and liver tissues were collected for further analysis. The sh*Gk* sequence is 5′‐GCTATAGCTGGTGCTGTAATC‐3′, and the shCon sequence is 5′‐CCTAAGGTTAAGTCGCCCTCGC‐3′. Ad‐Con, Ad‐Gk, shGk, and shCon were constructed by OBiO Technology Co. (Shanghai, China).

### Blood and Liver Biochemical Analyses

Serum levels of nonesterified fatty acids were measured using the NEFA assay kit (633‐52001, Wako Chemicals Inc., Richmond, USA). Serum levels of glycerol, aspartate transaminase, and alanine transaminase were determined using the Liquid Sample Glycerol Content Assay Kit (E1002, Applygen, China), the Glutamic‐oxalacetic Transaminase Assay Kit (AK087, Bioss, China), and the Glutamic‐pyruvic Transaminase Assay Kit (AK085, Bioss), respectively. Serum triglyceride (TG) concentrations, intracellular TG, and liver TG were measured using the Triglyceride Assay Kit (A110‐2‐1, Jiancheng Bioengineering Institute, China).

### H&E and Oil Red O Staining

The liver samples were fixed in 4% paraformaldehyde overnight at 4 °C. They were either embedded in paraffin or frozen at −15 °C and then sectioned. The sections were subjected to hematoxylin and eosin (H&E) staining and Oil Red O staining using the H&E staining kit (C0105S) and Oil Red O staining kit (C0157S) from Beyotime (Shanghai, China)) following the manufacturer's instructions. Images were captured using a light microscope.

### Immunofluorescence Staining

The expression of GK and SREBP‐1c in Huh7 cells was detected by immunofluorescence staining. Briefly, Huh7 cells cultured on glass coverslips were fixed in 4% paraformaldehyde for 15 min, permeabilized with 0.3% Tirton for 30 min, and blocked with 5% BSA for 60 min, followed by incubation with anti‐GK (ab228615, Abcam) and anti‐SREBP‐1c (sc‐13551, Santa Cruz Technology) antibodies, overnight at 4 °C, washed with Tris Buffered Saline with Tween‐20 (TBST) and incubated with Alexa Fluor 488 (A‐11001, Invitrogen) and Alexa Fluor 546 (A‐11035, Invitrogen) conjugated secondary antibodies for 1 h at room temperature. Cells were then stained with DAPI (10 236 276 001, Roche) for 15 min, washed with TBST, and mounted with Fluoromount (F4680, Sigma‐Aldrich). Images were captured with a laser scanning confocal microscope (Carl Zeiss LSM880).

### Plasmid Constructs

To construct the plasmids expressing full‐length human GK, the N‐terminal fragment of GK (amino acids 1–271, GK‐N), the C‐terminal fragment of GK (amino acids 272–524, GK‐C), or mouse Gk, RNA was isolated from HepG2 or AML12 cells and reverse transcribed into cDNA. The target cDNA was amplified by PCR and inserted into the pCMV‐Tag2B vector using the ClonExpress Ultra One Step Cloning Kit (C112, Vazyme Biotech Co., China). To generate plasmids that express mutant GKs (GK(D198G), GK(C256R), GK(N288D)), point mutations were introduced into the GK‐expression plasmid using the QuickMutation Site‐Directed Mutagenesis Kit (D0206M, Beyotime). To generate the human SREBP‐1c promoter‐luciferase reporter construct, the nucleotides spanning from −2000 to −1 upstream of the human SREBP‐1c gene from the genomic DNA of Huh7 cells by PCR was amplified. To create mouse Srebp‐1c promoter‐luciferase reporter constructs, the nucleotides spanning from −117 to +42, −278 to −118, −278 to +42, −574 to −279, and −574 to +42 of the mouse Srebp‐1c gene were amplified from the genomic DNA of AML12 cells by PCR. After gel purification, the PCR products were inserted into a pGL3‐basic vector (E1751, Promega, USA). The human SREBP‐1c and mouse Srebp‐1c coding regions were cloned into the p3×Flag‐CMV‐14 vector to construct the SREBP‐1c and Srebp‐1c expression plasmids, respectively. All plasmids were confirmed by DNA sequencing. PCR primers used to amplify cDNAs for GK, GK‐N, GK‐C, SREBP‐1c, Gk, and Srebp‐1c, to amplify nucleotides upstream of the mouse and human Srebp‐1c genes, are listed in Table S1 (Supporting Information).

### Cell Culture and Treatment

Huh7, HepG2, HEK 293T, and AML12 cells were obtained from the Cell Bank of the Chinese Academy of Sciences. Huh7, HepG2, and HEK 293T cells were cultured in Dulbecco's modified Eagle's medium (11995065, 10099141, Gibco) supplemented with 10% fetal bovine serum (FBS) and 1% penicillin/streptomycin, AML12 cells were cultured in DMEM/F12 (1:1) medium (11320033, Gibco) supplemented with 10% FBS, ITS Liquid Media Supplement (I3146, Sigma‐Aldrich) and dexamethasone (D4902, Sigma‐Aldrich). These cells were cultured in a humidified incubator at 37 °C with 5% CO_2_. To investigate the regulation of GK expression in hepatocytes, Huh7 cells were treated with a control medium, cholesterol, palmitate, insulin, or glucose for different time periods. To investigate the involvement of transcription factors in palmitate‐ and cholesterol‐induced Gk expression, Huh7 cells were transfected with *EGR1* siRNA (siEGR1), *KLF5* siRNA (siKLF5), *SP1* siRNA (siSP1) or control siRNA (siCon) using Lipofectamine 3000 Transfection Reagent (L3000015, Invitrogen, San Diego, CA, USA) for 12 h followed by stimulation with 400 µM palmitate for 20 h or 50 µM cholesterol for 20 h. The siEGR1 sequence is 5′‐CCAUGGACAACUACCCUAATT‐3′, the siKLF5 sequence is 5′‐AAGCUCACCUGAGGACUCATT‐3′, the siSP1 sequence is 5′‐GCAACAUGGGAAUUAUGAATT‐3′. To study the effect of GK, GK fragments, and mutant GKs on SREBP‐1c and its target lipogenic gene expression, Huh7 cells were transduced with adenoviruses expressing GK (Ad‐GK) or vector (Ad‐Con), or transfected with GK siRNA (siGK) or control siRNA (siCon), and with a plasmid expressing GK, GK fragments, mutant GKs, or control vector using Lipofectamine 3000 Transfection Reagent (L3000015, Invitrogen). The expression of SREBP‐1c and its target lipogenic genes were examined at the mRNA and protein levels by RT‐qPCR and Western blot after 24 and 48 h, respectively. The siGK sequence is 5′‐GAUAAACAACUCUGCGAAUUUTT‐3′, the siCon sequence is 5′‐UUCUCCGAACGUGUCACGUTT‐3′. To investigate the effect of GK on TG synthesis and the involvement of SREBP‐1c in hepatocytes, HepG2 cells were transfected with SREBP‐1c siRNA (siSREBP‐1c) or control siRNA (siCon), or treated with 10 µM NDI‐091143 (S8878, Selleck, USA), 10 µM Firsocostat (S8893, Selleck), 10 µM C75 (S9819, Selleck) for 12 h. The cells then were transfected with GK expression plasmid or control vector for 36 to determine intracellular TG levels. The sequence of siSREBP‐1c is 5′‐GCUGAAUAAAUCUGCUGUCUUTT‐3′.

### RNA Extraction and Quantitative Real‐Time PCR

Total RNA was extracted from cells or liver tissue using Trizol reagent (15596018, Invitrogen). cDNA was synthesized from total RNA using the PrimeScript RT Reagent Kit (RR037A, Takara, Japan). Quantitative real‐time PCR (qPCR) was performed by using QuantStudio 6 (ThermoFisher, USA) with SYBR Green PCR Master Mix (4367659, Applied Biosystems, MA). The relative expression of the target mRNA was calculated using the 2^−ΔΔCT^ method and normalized to mouse 18s or human β‐actin mRNA levels. The primer sequences used for qPCR are listed in Table S2 (Supporting Information).

### Western Blotting and Co‐Immunoprecipitation Assays

Proteins were extracted from cells or liver tissue using RIPA buffer (89900, Thermo Fisher Scientific). Cytoplasmic and nuclear proteins were separated from cells using the Nuclear and Cytoplasmic Protein Extraction Kit (P0027, Beyotime). The protein concentration was determined using the BCA kit (P0009, Beyotime). The proteins were separated by SDS‐PAGE and transferred to PVDF membrane. The membranes were incubated with primary antibodies overnight and then with HRP‐conjugated secondary antibodies for 1 h. The target proteins were detected using the SuperSignal West Pico Chemiluminescent Substrate (34580, Thermo Fisher Scientific), quantified using ImageJ, and normalized to the β‐actin, GAPDH, or lamin B1 protein. The primary antibodies were GK (ab126599, Abcam), SREBP‐1c (sc‐13551, Santa Cruz Biotechnology), ACC1 (4190, Cell Signaling Technology), ACLY (A22273, Abclonal), FASN (3180, Cell Signaling Technology), ELOVL6 (A21094, Abclonal), SCD1(2794, Cell Signaling Technology), DGAT (A6857, Abclonal), lamin B (13435, Cell Signaling Technology), β‐actin (4970, Cell Signaling Technology), and GAPDH (2118, Cell Signaling Technology). To perform co‐immunoprecipitation of mouse SREBP‐1c and GK, human SREBP‐1c and GK, GK fragments or mutant GKs, HEK 293T cells were co‐transfected with Flag‐Srebp‐1c plasmid and Myc‐Gk plasmid, or Flag‐SREBP‐1c plasmid and plasmids for Myc‐tagged GK, GK fragments, or mutant GKs using Lipofectamine 3000 Transfection Reagent (L3000015, Invitrogen). After 48 h, cell lysates, cytoplasm, or nuclei were collected and incubated with antibodies against Myc (AE010, Abclonal) or Flag (AE092, Abclonal) overnight at 4 °C. The Pierce Protein A/G Magnetic Beads (88803, Thermo Fisher Scientific) were added and incubated for 2 h at 4 °C. The beads were washed with TBST and boiled in a loading buffer. The supernatant was collected for Western blot analysis using antibodies against Myc or Flag peptide.

### Fatty Acid Analysis by Liquid Chromatography‐Mass Spectrometry (LC‐MS)

HepG2 cells were transfected with siCon or siSREBP‐1c for 12 h, followed by transfection with GK expression plasmid or control vector for 36 h (1 mM [^13^C]‐acetate (CLM‐440‐5, Cambridge Isotope Laboratories) was added 12 h after transfection). Lipids were collected in 90% methanol + 0.3 M KOH and extracted with formic acid and hexane, after drying with nitrogen gas, the lipids were solubilized with acetonitrile/MeOH (1:1, v/v). Lipid samples were separated using a Vanquish UHPLC system (Thermo Fisher Scientific) and an Acquity UPLC BEH C18 column (2.1 × 100 mm, 1.75 µm particle size; Waters, Milford, MA, USA), and analyzed on a Q Exactive Plus mass spectrometer (Thermo Scientific, Waltham, MA, USA). Chromatographic conditions were as follows: injection sample volume 5 µL, column temperature 25 °C, flow rate 0.3 mL mi^−1^n. Solvent A was 60% acetonitrile with 0.02% HCOOH, and solvent B was acetonitrile and isopropanol in a 1:1 (vol/vol) ratio. The Q‐Exactive Plus mass spectrometer was operated in negative ion mode scanning from m/z 100–700 with a resolution of 140000 at m/z 200 (AGC target 3e6, Maximum IT 200 ms) and the following conditions: sheath gas flow rate, 35 arb; aux gas flow rate, 10 arb; spray voltage, 2.8 kV; capillary temperature, 320 °C; scan range, m/z 100–700 with a resolution of 140000 at m/z 200 (AGC target 3e6, Maximum IT 200 ms). Natural isotope abundance correction was applied using AccuCor.

### Luciferase Reporter Assays

Luciferase reporter assays were conducted to examine the effect of mouse GK on Srebp‐1c transcription and SREBP‐1c‐induced Srebp‐1c transcription, as well as the effect of human GK, GK fragments, and mutant GKs on SREBP‐1c transcription and SREBP‐1c‐induced SREBP‐1c transcription. HEK 293T cells were transfected with the plasmids indicated in the figures together with either the mouse Srebp‐1c promoter‐luciferase reporter construct or the human SREBP‐1c promoter‐luciferase reporter construct and Renilla luciferase reporter plasmid using Lipofectamine 3000 Transfection Reagent (L3000015, Invitrogen). The cells were harvested 24 h after transfection, and Firefly and Renilla luciferase activities were measured using the Dual‐Luciferase Reporter Assay System (E1960, Promega).

### Chromatin Immunoprecipitation Assay (ChIP‐qPCR)

The ChIP assay was performed using the BeyoChIP Enzymatic ChIP Assay Kit (P2083S, Beyotime) following the manufacturer's instructions. AML12 cells were transfected with either the Myc‐Gk plasmid, Flag‐Srebp‐1c plasmid, or the corresponding vectors for 48 h. Alternatively, AML12 cells were transfected with Srebp‐1c siRNA (siSrebp‐1c) or control siRNA (siCon) for 24 h, followed by transfection with the Myc‐Gk plasmid or control vector for 48 h. The cells were fixed with 1% formaldehyde, lysed with MNase, and sonicated to yield short DNA fragments. The DNA‐protein complexes were immunoprecipitated with non‐specific IgG, antibodies against Myc or Flag, and collected using protein A/G magnetic beads. The immunoprecipitated chromatin DNA fragments were quantified and the promoter regions of the mouse Srebp‐1c gene were amplified by real‐time PCR. The primer sequences used for qPCR are listed in Table S3 (Supporting Information). The siSrebp‐1c sequence is 5′‐GGAUUGCACAUUUGAAGACAUTT‐3′, and the siCon sequence is 5′‐CCUAAGGUUAAGUCGCCCUCGTT‐3′.

### Glycerol Kinase Activity Analysis

Huh7 cells were transfected with plasmids expressing GK, mutant GKs, or a control vector using Lipofectamine 3000 Transfection Reagent (Invitrogen, L3000015). Glycerol kinase activity was determined after 36 h using the Glycerol Kinase Activity Assay Kit (G0919F, Grace Biotechnology, China).

### Statistical Analysis

Each experiment was replicated at least three times. All data were presented as mean ± SEM. Statistical analysis was performed using GraphPad Prism 9 software (GraphPad Software, CA, USA). The sample size (*n*), probability (*P*) value, and statistical test for each experiment were detailed in each figure legend. The following symbols were used to indicate significance levels: * for *p <* 0.05, ** for *p <* 0.01, *** for *p <* 0.001.

## Conflict of Interest

The authors declare no conflict of interest.

## Author Contributions

Y.L., O.S., and S.Z. designed the research. O.S., S.Z., M.Y., and T.Z. performed most of the experiments and analyzed the data. S.Y. assisted in some experiments and data analysis. Y.L. and H.Y provided experimental materials. O.S. wrote the manuscript. Y.L. supervised the project and finalized the manuscript.

## Supporting information

Supporting Information

## Data Availability

The data that support the findings of this study are available from the corresponding author upon reasonable request.
