# Supplementary material for: Glycerol Kinase Drives Hepatic de novo Lipogenesis and Triglyceride Synthesis in Nonalcoholic Fatty Liver by Activating SREBP‐1c Transcription, Upregulating DGAT1/2 Expression, and Promoting Glycerol Metabolism
Source: Adv Sci (Weinh). 2024 Oct 17;11(46):2401311. doi: 10.1002/advs.202401311 (PMC11633478; doi:10.1002/advs.202401311)
Supplement: Supplementary file 1 — Supporting Information [file ADVS-11-2401311-s001.pdf]

ADVANCED SCIENCE
Open Access

Supporting Information

for *Adv. Sci.*, DOI 10.1002/advs.202401311

Glycerol Kinase Drives Hepatic de novo Lipogenesis and Triglyceride Synthesis in Nonalcoholic Fatty Liver by Activating *SREBP-1c* Transcription, Upregulating DGAT1/2 Expression, and Promoting Glycerol Metabolism

*Shuyu Ouyang, Shu Zhuo, Mengmei Yang, Tengfei Zhu, Shuting Yu, Yu Li, Hao Ying and Yingying Le**

Supporting Information

Glycerol kinase drives hepatic de novo lipogenesis and triglyceride synthesis in non-alcoholic fatty liver by activating *SREBP-1c* transcription, upregulating DGAT1/2 expression, and promoting glycerol metabolism

*Shuyu Ouyang, Shu Zhuo, Mengmei Yang, Tengfei Zhu, Shuting Yu, Yu Li, Hao Ying, and Yingying Le**

This file includes:

Supplementary Figures S1-S6

Table S1-S3

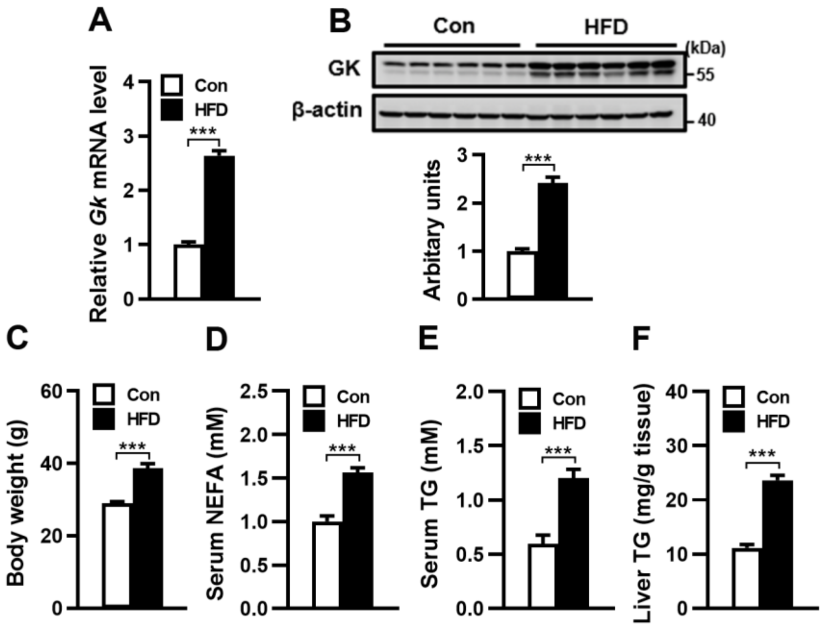

Figure S1. Effects of a high-fat diet on hepatic GK expression, body weight, blood and hepatic lipid levels in mice. Hepatic glycerol kinase (GK) at mRNA (A) and protein (B) levels, body weight (C), serum levels of nonesterified fatty acids (NEFA) (D) and triglyceride (TG) (E), as well as hepatic TG content (F) in mice fed either a control diet (Con) or a high-fat diet (HFD) for 2 months. n=6-8/group. Data are expressed as mean ± SEM. *p* values were calculated using an unpaired two-tailed Student's t test. ***$p < 0.001$.

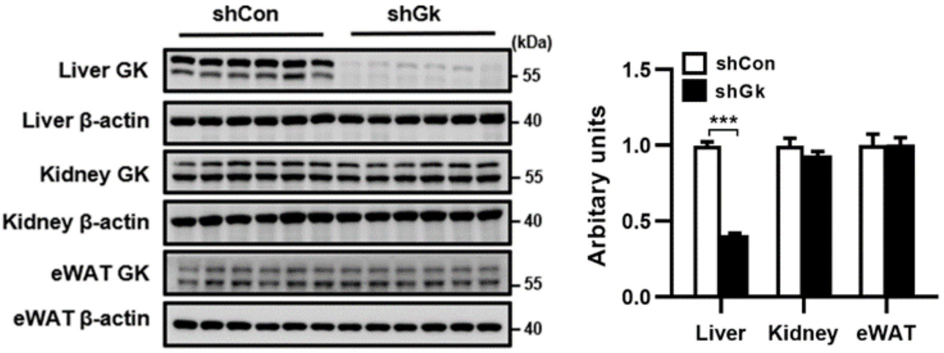

Figure S2. Knockdown of hepatic GK in HFD-fed mice by RNA interference. Mice fed with a high-fat diet (HFD) for 2 months were infected with adenoviruses expressing Gk shRNA (shGk) or control sequence (shCon) for 2 weeks. GK expression in liver, kidney, and epididymal white adipose tissue (eWAT) was examined by Western blot. n=6/group. Data are presented as mean ± SEM. p values were determined by unpaired two-tailed Student's t test. ***$p < 0.001$.

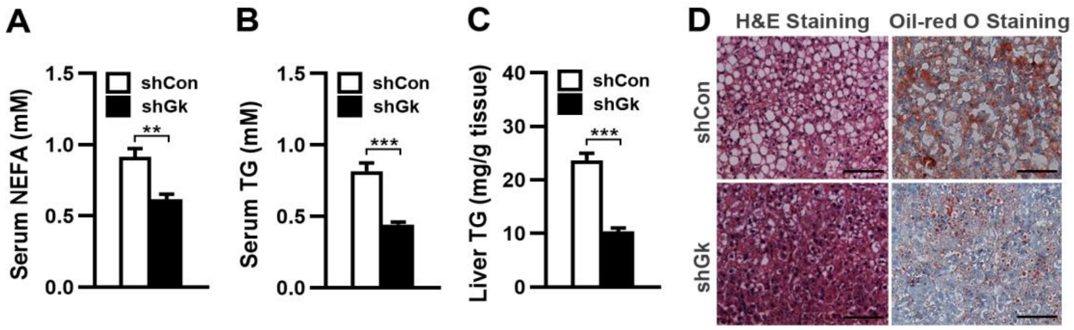

Figure S3. Knockdown of hepatic *Gk* in HFD-fed mice decreases serum lipid levels and reduces hepatic lipid deposition. Mice fed with a high-fat diet (HFD) for 3 months were infected with adenoviruses expressing Gk shRNA (shGk) or control sequence (shCon) for 2 weeks. Serum levels of nonesterified fatty acids (NEFA) (A) and triglyceride (TG) (B), as well as hepatic TG content (C) were examined. Hepatic lipid deposition was detected by H&E staining and Oil-red O staining (D). n=6-8/group. Data are presented as mean ± SEM. *p* values were determined using an unpaired two-tailed Student's t test. **$p < 0.01$, ***$p < 0.001$. Scale bar: 100 μm.

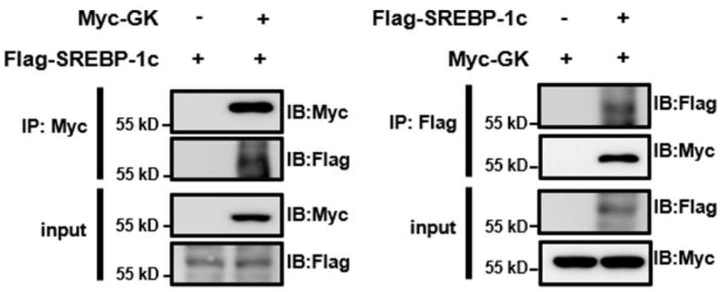

Figure S4. Co-immunoprecipitation of human GK and SREBP-1c protein in HEK 293T cells. HEK293T cells were transfected with plasmid for Myc-GK, Flag-SREBP-1c or both. Co-immunoprecipitation was performed using antibodies against Myc or Flag, followed by Western blot to detect GK and SREBP-1c using anti-Myc and anti-Flag antibodies, respectively.

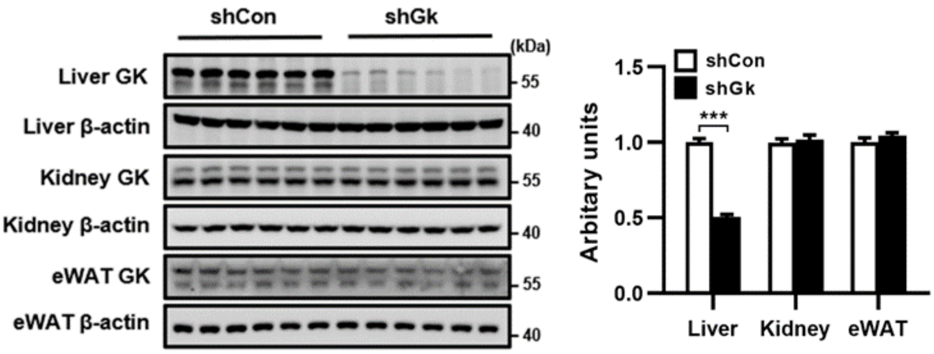

Figure S5. Knockdown of hepatic GK in normal chow-fed mice by RNA interference. Mice fed with normal chow for 2 months were infected with adenoviruses expressing Gk shRNA (shGk) or control sequence (shCon) for 2 weeks. GK expression in liver, kidney and epididymal white adipose tissue (eWAT) was examined by Western blot. n=6/group. Data are presented as mean ± SEM. p values were determined by unpaired two-tailed Student's t test. ***$p < 0.001$.

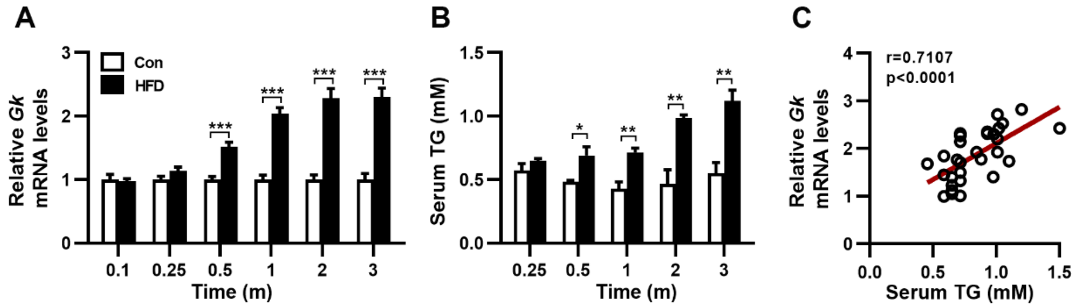

Figure S6. Association between hepatic Gk mRNA and serum TG levels in high-fat diet-fed mice. (A, B) Hepatic *Gk* mRNA (A) and serum TG (B) levels in mice fed a high-fat diet (HFD) for different periods of time. n=6/group. Data are presented as mean ± SEM. p values were determined by unpaired two-tailed Student's t test. $*p < 0.05$, $**p < 0.01$, $***p < 0.001$. (C) Spearman correlation analysis between hepatic Gk mRNA levels and serum TG levels in HFD-fed mice.

(Figure B is taken from our previously published paper (Figure S1E, Mol Nutr Food Res. 2021 Nov;65(21):e2100220). Gk mRNA expression was examined in liver tissue from these animals. The publisher WILEY allows authors to access and reuse their own work).

Table S1. Primer sequences for molecular cloning of genes

Plasmids	PCR primer sequences (5'→3')
Tag2B-Myc-Gk	F：GATCTTAGCCCGGGCGGATCCATGGCAGCCGCGAAGAAAGCA<br>R：GTCGACGGTATCGATAAGCTTTTATGGGATACCACTTTCTGG
Tag2B-Myc-GK	F：AGGAATTCGATATCAAGCTTATGGCAGCCTCAAAGAAGGCAG<br>R：GCCCCCCCTCGAGGTCGACTTATGGAATACCACTTTCTGGAG
Tag2B-Myc-GK-C (272-524)	F：AGGAATTCGATATCAAGCTTATGATTGGACAAGCCAAAAATAC<br>R：GGCCCCCCCTCGAGGTCGACTTATGGAATACCACTTTCTGGAG
Tag2B-Myc-GK-N (1-271)	F：AGGAATTCGATATCAAGCTTATGGCAGCCTCAAAGAAGGCAG<br>R：CCCTCGAGGTCGACTTACTGGAAGCACATTTGTCCCACCAAT
3×Flag-CMV-Srebp-1c	F：GAATTAAGCTTGCGGCCGCATGGATTGCACATTTGAAGACAT<br>R：TGTAGTCAGCCCGGGATCCCTAGCTGGAAGTGACGGTGGTTC
3×Flag-CMV-SREBP-1c	F：AGAATTAAGCTTGCGGCCGCATGGATTGCACTTTCGAAGACA<br>R：TTGTAGTCAGCCCGGGATCCCTAGCTGGAAGTGACAGTGGTC
Tag2B-Myc-GK(D198G)	F：GAGGTGTCCACTGTACAGGTGTAACAAATGCAAGTAG<br>R：CTACTTGCATTTGTTACACCTGTACAGTGGACACCTC
Tag2B-Myc-GK(C256R)	F：GTGCCAATATCTGGGCGTTTAGGGGACCAGT<br>R：ACTGGTCCCCTAAACGCCCAGATATTGGCAC
Tag2B-Myc-GK(N288D)	F：CAGGATGTTTCTTACTATGTGATACAGGCCATAAGTGTG<br>R：CACACTTATGGCCTGTATCACATAGTAAGAAACATCCTG
pGL3-SREBP-1c-Luc	F：CTCTATCGATAGGTACCGGTGAAACCTCATCTCTACTAA<br>R：GTACCGGAATGCCAAGCTTGGGTTCCCCCGGCCGCAGCTC
pGL3-Srebp-1c (-574/+42)- Luc	F：CTCTATCGATAGGTACCGGATCCAGAACTGGATCATCAG<br>R：GTACCGGAATGCCAAGCTTCCTAGGGCGTGCAGACGCTA
pGL3-Srebp-1c (-117/+42)- Luc	F：CTCTATCGATAGGTACCGGGCGGGGCCCTAATGGGGC<br>R：GTACCGGAATGCCAAGCTTCCTAGGGCGTGCAGACGCTA
pGL3-Srebp-1c (-278/-118)- Luc	F：CTCTATCGATAGGTACCCTTTTCGGGGATGGTTGCCTGTG<br>R：GTACCGGAATGCCAAGCTTACCCCCGACGGCGGCAGCTC
pGL3-Srebp-1c (-428/-279)- Luc	F：CTCTATCGATAGGTACCCTTTTCGGGGATGGTTGCCTGTG<br>R：GTACCGGAATGCCAAGCTTCCTAGGGCGTGCAGACGCTA
pGL3-Srebp-1c (-574/-429)- Luc	F：CTCTATCGATAGGTACCGGATCCAGAACTGGATCATCAGC<br>R：GTACCGGAATGCCAAGCTTAGCCTGGGTCCGGAATCCGGA

Table S2. Primer sequences for RT-qPCR

Genes	Primer sequences (5’→3)
Mouse 18s	F: CCCTGCCCTTTGTACACACC<br>R: CGATCCGAGGGCCTCACTA
Mouse *Gk*	F: TGAACCTGAGGATTTGTCAGC<br>R: CCATGTGGAGTAACGGATTTCG
Mouse *Srebp-1c*	F: TGACCCGGCTATTCCGTGA<br>R: CTGGGCTGAGCAATACAGTTC
Mouse *Acc1*	F: ATGGGCGGAATGGTCTCTTTC<br>R: TGGGGACCTTGTCTTCATCAT
Mouse *Acly*	F: ACCCTTTCACTGGGGATCACA<br>R: GACAGGGATCAGGATTTCCTTG
Mouse *Fasn*	F: GGAGGTGGTGATAGCCGGTAT<br>R: TGGGTAATCCATAGAGCCCAG
Mouse *Elovl6*	F: GAAAAGCAGTTCAACGAGAACG<br>R: AGATGCCGACCACCAAAGATA
Mouse *Scd1*	F: TTCTTGCGATACACTCTGGTGC<br>R: CGGGATTGAATGTTCTTGTCGT
Mouse *Dgat1*	F: TCCGTCCAGGGTGGTAGTG<br>R: TGAACAAAGAATCTTGCAGACGA
Mouse *Dgat2*	F: GCGCTACTTCCGAGACTACTT<br>R: GGGCCTTATGCCAGGAAACT
Human *Actin*	F: CATGTACGTTGCTATCCAGGC<br>R: CTCCTTAATGTCACGCACGAT
Human *GK*	F: GAACCCAGTCTACCGTTGAGA<br>R: TGGACACCTCCATTGACTCCT
Human *SREBP-1c*	F: ACAGTGACTTCCCTGGCCTAT<br>R: GCATGGACGGGTACATCTTCAA
Human *ACC1*	F: TCACACCTGAAGACCTTAAAGCC<br>R: AGCCCACACTGCTTGTACTG
Human *ACLY*	F: ATCGGTTCAAGTATGCTCGGG<br>R: GACCAAGTTTTCCACGACGTT
Human *FASN*	F: ACAGCGGGGAATGGGTACT<br>R: GACTGGTACAACGAGCGGAT
Human *ELOVL6*	F: AACGAGCAAAGTTTGAACTGAGG<br>R: TCGAAGAGCACCGAATATACTGA
Human *SCD1*	F: TTCCTACCTGCAAGTTCTACACC<br>R: CCGAGCTTTGTAAGAGCGGT
Human *DGAT1*	F: CAATCTGACCTACCGCGATCT<br>R: TCGATGATGCGTGAGTAGTCC
Human *DGAT2*	F: GAATGGGAGTGGCAATGCTAT<br>R: CCTCGAAGATCACCTGCTTGT

Table S2. Primer sequences for RT-qPCR (continued)

Genes	Primer sequences (5'→3)
Human *EGR1*	F: CCACGCCGAACACTGACATT<br>R: GAGGGGTTAGCGAAGGCTG
Human *KLF5*	F: ACACCAGACCGCAGCTCCA<br>R: TCCATTGCTGCTGTCTGATTTGTAG
Human *SP1*	F: CCACCATGAGCGACCAAGAT<br>R: GTAGCCCCAGAGGAGGAAGA

Table S3. Primer sequences for ChIP-qPCR

Srebp-1c promoter region	Primer sequences (5'→3)
-117/+42	F: GGGCGGGGCCCTAATGGGGC<br>R: CCTAGGGCGTGCAGACGCTA
-278/-118	F: CTTTTCGGGGATGGTTGCCTGTGCG<br>R: ACCCCCGACGGCGGCAGC
-428/-279	F: CTGCTGCCATTCGATGCGAAGGGCC<br>R: AGCCTGGGTCCGGAATCCGGACG
-574/-429	F: GGATCCAGAACTGGATCATCAGCC<br>R: CAAGATTTGCCTACAGTCTGAGGAAG
